# Supplementary material for: Household food sources and diarrhoea incidence in poor urban communities, Accra Ghana
Source: PLoS One. 2021 Jan 28;16(1):e0245466. doi: 10.1371/journal.pone.0245466 (PMC7842991; doi:10.1371/journal.pone.0245466)
Supplement: S1 File — (ZIP) [file pone.0245466.s001.zip › questionnaires and dataset - diarrhoea and food sources/Wave 3 - RIPS HOUSEHOLD QUESTIONNAIRE.pdf]

REGIONAL INSTITUTE FOR POPULATION STUDIES
UNIVERSITY OF GHANA

POPULATION TRAINING AND RESEARCH CAPACITY FOR DEVELOPMENT (POPTRCD)

URBAN HEALTH AND POVERTY PROJECT

HOUSEHOLD QUESTIONNAIRE

START TIME FOR INTERVIEW	HOURS MINS

IDENTIFICATION

LOCALITY NAME* ________________	
E.A. BASE ________________	
NAME OF HOUSEHOLD HEAD ________________	
E.A. /EDL NUMBER	
STRUCTURE NUMBER..	
HOUSEHOLD NUMBER	
GREATER ACCRA..	0 3
ROUND..	0 3
* **CODES FOR LOCALITY NAME:** 1=AGBOGBLOSHIE 2=JAMES TOWN 3=USSHER TOWN	

INTERVIEWER VISITS

	1	2	3	FINAL VISIT
DATE	________	________	________	DAY<br>MONTH<br>YEAR 2 0 1 3
INTERVIEWER'S NAME	________	________	________	INT. CODE
RESULT*	________	________	________	RESULT
Next visit: Date	________	________		TOTAL NO. OF VISITS
Time	________	________		

***RESULT CODES:**<br>1 COMPLETED<br>2 PARTLY COMPLETED<br>3 NO HOUSEHOLD MEMBER AT HOME OR NO COMPETENT RESPONDENT AT HOME AT THE TIME OF VISIT<br>4 ENTIRE HOUSEHOLD ABSENT FOR EXTENDED PERIOD OF TIME<br>5 POSTPONED<br>6 REFUSED<br>7 DWELLING VACANT OR ADDRESS NOT A DWELLING<br>8 DWELLING DESTROYED<br>9 DWELLING NOT FOUND<br>10 OTHER (SPECIFY) ________________	TOTAL PERSONS IN HOUSEHOLD<br>TOTAL ELIGIBLE WOMEN<br>TOTAL ELIGIBLE MEN<br>LINE NO. OF RESP. TO HOUSEHOLD QUEST.<br>HH INT. IN ROUND 2 1=YES 2=NO

LANGUAGE

LANGUAGE OF QUESTIONNAIRE: **ENGLISH**	1
LANGUAGE OF INTERVIEW** ________________	
NATIVE LANGUAGE OF RESPONDENT** ________________	
WAS TRANSLATOR USED? (YES=1, NO=2)	
****LANGUAGE CODES**:<br>1= ENGLISH 2 =AKAN 3= GA 4 =EWE 5 =DAGBANI<br>6= HAUSA 7= OTHER (SPECIFY) ________________	

SUPERVISOR	FIELD EDITOR	KEYED BY
NAME ________<br>DATE ________	NAME ________<br>DATE ________	

HOUSEHOLD SCHEDULE

I would like some information about the people who usually live in your household or who are staying with you now.

LINE NO.	USUAL RESIDENTS AND VISITORS	RELATION-SHIP TO HEAD OF HH	SEX	RESIDENCE			AGE	ELIGIBILITY		
	Please give me the names of the persons who usually live in your household and guests of the household who stayed here last night, starting with the head of the household.	What is the relationship of (NAME) to the head of the household?*	Is (NAME) male or female?	Does (NAME) usually live here? (6months or more)	Did (NAME) sleep here last night? **YES**→8	Why did (NAME) not sleep here last night?**	How old is (NAME)?	CIRCLE LINE NUMBER OF ALL CHILDREN UNDER AGE 5	CIRCLE LINE NUMBER OF ALL WOMEN AGE 15-49***	CIRCLE LINE NUMBER OF ALL MEN AGE 15-59***
(1)	(2)	(3)	(4)	(5)	(6)	(7)	(8)	(9)	(10)	(11)
01			M F 1 2	YES NO 1 2	YES NO 1 2		IN YEARS	01	01	01
02			M F 1 2	YES NO 1 2	YES NO 1 2		IN YEARS	02	02	02
03			M F 1 2	YES NO 1 2	YES NO 1 2		IN YEARS	03	03	03
04			M F 1 2	YES NO 1 2	YES NO 1 2		IN YEARS	04	04	04
05			M F 1 2	YES NO 1 2	YES NO 1 2		IN YEARS	05	05	05
06			M F 1 2	YES NO 1 2	YES NO 1 2		IN YEARS	06	06	06
07			M F 1 2	YES NO 1 2	YES NO 1 2		IN YEARS	07	07	07
08			M F 1 2	YES NO 1 2	YES NO 1 2		IN YEARS	08	08	08
09			M F 1 2	YES NO 1 2	YES NO 1 2		IN YEARS	09	09	09
10			M F 1 2	YES NO 1 2	YES NO 1 2		IN YEARS	10	10	10
11			M F 1 2	YES NO 1 2	YES NO 1 2		IN YEARS	11	11	11
12			M F 1 2	YES NO 1 2	YES NO 1 2		IN YEARS	12	12	12
13			M F 1 2	YES NO 1 2	YES NO 1 2		IN YEARS	13	13	13
14			M F 1 2	YES NO 1 2	YES NO 1 2		IN YEARS	14	14	14
15			M F 1 2	YES NO 1 2	YES NO 1 2		IN YEARS	15	15	15

TICK HERE IF CONTINUATION SHEET USED ☐ **NUMBER OF ELIGIBLE WOMEN** ☐☐ **NUMBER OF ELIGIBLE MEN** ☐☐

*** CODES FOR Q3**
RELATIONSHIP TO HEAD OF HOUSEHOLD:

01 = HEAD
02 = SPOUSE
03 = SON OR DAUGHTER
04 = SON-IN-LAW/DAUGHTER-IN-LAW
05 = GRANDCHILD
06 = PARENT
07 = PARENT-IN-LAW
08 = BROTHER/SISTER
09 = CO-WIFE
10 = ADOPTED/FOSTER/STEP-CHILD
11= OTHER RELATIVE (AFFINAL)
12= OTHER RELATIVE (CONSANGUINE)
13= NOT RELATED
98=DON'T KNOW

****CODES FOR Q7**

01=AT WORK
02=NO SPACE FOR SLEEPING
03=TRAVELLED
04=BOARDING HOUSE
06=OTHER (SPECIFY)……….
98=DON'T KNOW

LINE NO.		EDUCATION (IF AGE 3 OR OLDER & IF ATTENDED SCHOOL)			IF AGE 15 OR OLDER MARITAL STATUS	ETHNICITY	RELIGION
	Has (NAME) ever attended school? **NO→16**	What is the highest level of education (NAME) attended?*	What is the highest grade (NAME) completed at that level?**	Is (NAME) still in school?	What is the marital status of (NAME)? ***	What is the ethnic group of (NAME)? ****	What is the religion of (NAME)? *****
	**(12)**	**(13)**	**(14)**	**(15)**	**(16)**	**(17)**	**(18)**
01	YES NO<br>1 2			YES NO<br>1 2		96=OTHER SPECI	
02	YES NO<br>1 2			YES NO<br>1 2		96=OTHER SPECIF	
03	YES NO<br>1 2			YES NO<br>1 2		96=OTHER	
04	YES NO<br>1 2			YES NO<br>1 2		96=OTHER SPECIFY	
05	YES NO<br>1 2			YES NO<br>1 2		96=OTHER SPECIFY	
06	YES NO<br>1 2			YES NO<br>1 2		96=OTHER SPECIFY	
07	YES NO<br>1 2			YES NO<br>1 2		96=OTHER SPECIFY	
08	YES NO<br>1 2			YES NO<br>1 2		96=OTHER SPECIFY	
09	YES NO<br>1 2			YES NO<br>1 2		96=OTHER SPECIFY	
10	YES NO<br>1 2			YES NO<br>1 2		96=OTHER SPECIFY	
11	YES NO<br>1 2			YES NO<br>1 2		96=OTHER SPECIFY	
12	YES NO<br>1 2			YES NO<br>1 2		96=OTHER SPECIFY	
13	YES NO<br>1 2			YES NO<br>1 2		96=OTHER SPECIFY	
14	YES NO<br>1 2			YES NO<br>1 2		96=OTHER SPECIFY	
15	YES NO<br>1 2			YES NO<br>1 2		96=OTHER SPECIF.	

Just to make sure that I have a complete listing:

1) Are there any other persons such as infants or children that we have not listed? YES → ENTER EACH IN TABLE NO
2) In addition, are there any other people such as domestic servants, lodgers or friends who usually live here? YES → ENTER EACH IN TABLE NO
3) Are there any guests or temporary visitors staying here, or anyone else who slept here last night that I have not listed? YES → ENTER EACH IN TABLE NO (SKIP ONE ROW)
4) Are there any persons who used to live in your household but have moved out in the past 1 year? YES → ENTER EACH IN TABLE NO HOW MANY?

***CODES FOR Q13**
0=PRE-SCHOOL
1=PRIMARY
2=JHS/MIDDLE
3=SHS/SECONDARY
4=HIGHER
8=DON'T KNOW

****EDUCATION GRADE Q14**
00=LESS THAN 1 YEAR
98=DON'T KNOW

*****CODES FOR Q16**
0=NEVER MARRIED
1= LIVING TOGETHER
2= MARRIED
3= SEPARATED
4=DIVORCED
5= WIDOWED

******CODES FOR Q17**
01=AKAN
02=GA-DANGME
03=EWE
04=GUAN
05=GURMA
06=MOLE-DAGBANI
07=GRUSI
08=MANDE
96=OTHER (SPECIFY)
RECORD ADJACENT TO TO THE CODE ABOVE.

*******CODES FOR Q18**
01= NO RELIGION
02= CATHOLIC
03= PROTESTANTS
04= PENTECOSTAL/CHARISMATIC
05=OTHER CHRISTIAN
06=ISLAM
07=TRADITIONAL/SPIRITUALIST
08=EASTERN RELIGIONS (ECKANKA , HINDU)
96=OTHER (SPECIFY)..

LINE NO.	FOR VISITORS AND USUAL RESIDENTS	VISITORS ONLY	FOR FORMER RESIDENTS/VISITORS ONLY	OCCUPATION	PopTRCD 2011
	**FOR VISITORS:** How long has (NAME) been staying here?** **FOR USUAL RESIDENTS** How long has (NAME) lived in this household? ****(USE CODES BELOW)****	Where did (NAME) come from? **USE CODES BELOW)***	**ONLY IF '2' IS CIRCLED IN BOTH COLS. 5 & 6** How long did (NAME) live here before moving out?**	What is (NAME) current occupation? That is what kind of work does (NAME) mainly do?	Was (NAME) a member of this household during the **2011 EDUL (Nov./Dec.)** survey? 1=YES→ **Q28** 2=NO 8= DON'T KNOW
	(19)	(20)	(21)	(22)	(23)
01					
02					
03					
04					
05					
06					
07					
08					
09					
10					
11					
12					
13					
14					
15					

*CODES FOR Q20	**CODES FOR Q19 AND Q21	***CODES FOR Q22	
1= WITHIN THE SAME COMMUNITY	1= DAY	01=NO OCCUPATION	07=AGRICULTURE
2= ANOTHER COMMUNITY IN ACCRA	2= WEEKS	02=PROFESSIONAL/TECHNICAL	08=HOUSEHOLD AND DOMESTIC
3= ANOTHER TOWN	3=MONTHS	03=MANAGEMENT	09=SERVICE
4= RURAL	4= YEARS	04=CLERICAL	10=SKILLED MANUAL
5= BOARDING SCHOOL	5=SINCE BIRTH	05=SALES	11=UNSKILLED MANUAL
8=DON'T KNOW	8=DON'T KNOW	06=AGRICULTURE- SELF EMPLOYED	12=STUDENT
		98=DON'T KNOW	96=OTHER (SPECIFY)...............

	(24)	(25)	(26)			(27)		
	Was there any person(s) who was a member of your household during **2011 PopTRCD (EDULINK)** survey but currently not a member now? **1=YES 2=NO → SKIP TO Q28**	Please give me the name(s) of that person(s)	What is the reason for [NAME]'s absence?****			Where did [NAME] go to?*****		
		a.						
		b.						
		c.						
		d.						

****CODES FOR Q26		*****CODES FOR Q27
01 = EMPLOYMENT	07 = PERSONAL REASONS	1 = DIFFERENT HOUSEHOLD IN SAME COMMUNITY/LOCALITY/ NEIGHBOURHOOD
02 = LOOKING FOR WORK	08 = ESCAPE VIOLENCE OR POLITICAL PROBLEMS	2 = RURAL AREA IN DIFFERENT PART OF THE COUNTRY
03 = SCHOOL	09 = PRISON	3 = CITY/URBAN AREA IN DIFFERENT PART OF THE COUNTRY
04 = VISIT FAMILY	10 = HOSPITAL /CLINIC	4 = ANOTHER COUNTRY 8 = DON'T KNOW
05 = VISIT FRIENDS	11 = NURSING HOME/OLD PERSONS HOME	
06 = MARRIAGE/COHABITATION	12 = DIED _ go to Next HH member	
	96= OTHER (SPECIFY)..........................	

CHRONIC NON-COMMUNICABLE DISEASE CONDITIONS

LINE NO.	Has (Name) ever been told by a health professional that he/she has had a **stroke**?<br>1. YES<br>2. NO → **Q31**<br>***If less than 1 year for Q28b, code 00***		Has (Name) been taking any medications or other treatment for it during **the last 2 weeks?**<br>1. YES<br>2. NO	Has (Name) been taking any medications or other treatment for it during **the last 12 months?**<br>1. YES<br>2. NO	Has (Name) ever been diagnosed with high blood pressure (**hypertension**)? ***(Not including hypertension associated with a pregnancy)***<br>1. YES<br>2. NO → **Q34**<br>***If less than 1 year for Q31b, code 00***		Has (Name) been taking any medications or other treatment for it during **the last 2 weeks?**<br>1. YES<br>2. NO<br>**(Other treatment might include weight loss program or change in eating habits.)**	Has (Name) been taking any medications or other treatment for it during **the last 12 months**?<br>1. YES<br>2. NO<br>**(Other treatment might include weight loss program or change in eating habits.)**
	**(28a)**	**(28b)**<br>**IF YES, HOW LONG(years)?**	**(29)**	**(30)**	**(31a)**	**(31b)**<br>**IF YES, HOW LONG(years)?**	**(32)**	**(33)**
01								
02								
03								
04								
05								
06								
07								
08								
09								
10								
11								
12								
13								
14								
15								

CHRONIC NON-COMMUNICABLE DISEASE CONDITIONS

LINE NO.	Has (Name) ever been diagnosed with **diabetes** (high blood sugar)? (Not including diabetes associated with a pregnancy)<br>1. YES<br>2. NO ➔**Q37**<br>***If less than 1 year for Q34b, code 00***		Has (Name) been taking insulin or other blood sugar lowering medications during **the last 2 weeks**?<br>1. YES<br>2. NO	Has (Name) been taking insulin or other blood sugar lowering medications during **the last 12 months?**<br>1. YES<br>2. NO	Has (Name) ever been diagnosed with any other chronic non-communicable disease apart from conditions mentioned in **(Q28, Q31, and Q34 - Stroke, hypertension and diabetes)**<br>1. YES 2. NO ➔**Q41**	If Yes in **Q37**, please specify:<br>***(Interviewer, record all mentioned)****<br>***If less than 1 year for Q34b, code 00***		Has (Name) been taking any medication or therapy for the condition during **the last 2 weeks**?<br>1. YES<br>2. NO	Has (Name) been taking any medication or therapy for the condition during **the last 12 months?**<br>1. YES<br>2. NO
	**(34a)**	**(34b) IF YES, HOW LONG (years)?**	**(35)**	**(36)**	**(37)**	**(38a)**	**(38b) IF YES, HOW LONG (years)?**	**(39)**	**(40)**
01									
02									
03									
04									
05									
06									
07									
08									
09									
10									
11									
12									
13									
14									
15									

Code For Q38*
1=ARTHRITIS
2=ANGINA (coronary heart disease)
3=ASTHMA
4=CANCER
5=DEPRESSION
6= OTHER (SPECIFY)……………………………….

HOUSEHOLD CHARACTERISTICS

NO.	QUESTION	RESPONSE
41	What is the **main** source of water supply for your household?<br>01=INDOOR PLUMBING<br>02=PRIVATE OUTSIDE STANDPIPE/TAP<br>03=RIVER/STREAM /SPRING<br>04=INSIDE STANDPIPE<br>05=PUBLIC STANDPIPE<br>06= RAIN WATER<br>07=WATER TRUCK/TANKER<br>08=BOLEHOLE<br>09=DUGOUT/POND/LAKE/DAM<br>10=WATER VENDOR<br>11=PROTECTED WELL<br>12=PIPE IN NEIGHBORING HOUSEHOLD<br>13=UNPROTECTED WELL<br>14=SACHET/BOTTLED WATER<br>15=OTHER (SPECIFY)_____________	
42	How much water does your household use in a day?	

UNIT	QUANTITY
Litres (L)	
Gallons (G)	
Buckets (B)	
Other (specify) (O)………….	

43 Please complete the following table relating to your household water use **(by the individuals who fetch water)**

**Individual**<br><br>**(LINE NO.)**	**Estimated hourly income/wage**<br><br>**Amount in GH₵**	**Water source**<br><br>***Use Code in Q 41***	**Round trip time to water source**<br><br>**(minutes)**	**Time spent at the source**<br><br>**(minutes)**	**Quantity of water collected per trip** **L**	**G**	**B**	**0**	**Price per quantity indicated**<br><br>**Amount in GH ₵**	**Number of trips per week**

NO.	QUESTION	RESPONSE	
44	How regular is the flow of water from your **main** source of water supply?<br>1=DAILY 2=WEEKLY 3=FORTNIGHTLY 4=MONTHLY 5=Other (specify)………………….		
45	How is the main water source system managed/operated?<br>1= SELF<br>2=COMMUNITY OPERATED AND MANAGED<br>3=NGO<br>4=COMMUNITY WATER AND SANITATION AGENCY<br>5=GHANA WATER COMPANY LTD<br>6=OTHER SPECIFY		
46	If you have an indoor plumbing system, does the household pay a regular (monthly) bill for this water supply? 1=YES 2=NO 3=NO INDOOR PLUMBING		**If code 2 or 3 skip to 48**
47	How much was the last bill? (Only your part if joint meter or shared bill)	Amount in GH₵	
48	How much did your household pay to a private water vendor, water from neighbouring standpipe, or any other source in the last 2 weeks	Amount in GH₵	
49	Did your household sell any water to someone else?<br>1=YES 2=NO		**If code 2, skip to 51**
50	How much did your household receive from the water sold in the last 2 weeks?<br>Don't know=999.98	Amount in GH₵	
51	Do you store water in your house so as to use it for more than one day?<br>1=YES 2=NO		**If code 2, skip to 57**

NO	Question	Response	SKIP
52	How do you **mainly** store your drinking water? *Codes below*		IF CODE , 1,3,5,7,8 9 SKIP to 55
53	If container has a lid, does the lid screw on or attach tightly to the container?<br>1=YES 2=NO		
54	Does the container have a spigot or small mouth or tap for dispensing water?<br>1=YES 2=NO		
55	Do you do anything to make this water safe to drink?<br>1=YES 2=NO		IF CODE 2 OR 8, SKIP to 57
56	What do you usually do to make the water safe to drink? *Codes below*		
57	What kind of toilet facility does your household **mainly** use?<br>*Codes below*		IF CODE 1, SKIP to 59
58	Do you share the above mentioned toilet facility with other households?<br>1=YES 2=NO		
59	Do you have a refuse bin in your household?<br>1=YES 2=NO		
60	Who **usually** disposes of the household solid waste?<br>1=ADULT WOMAN 2=ADULT MAN<br>3=FEMALE CHILD (UNDER 15 YEARS) 4=MALE CHILD (UNDER 15 YEARS)		
61	Where do you dispose of household solid waste?<br>01=COLLECTED AT HOME BY A PRIVATE COMPANY 05=TRUCK PUSHERS (KAYA BOLA<br>02=COLLECTED AT HOME BY A GOVERNMENT AGENCY 06=INDISCRIMINATELY<br>03=REFUSE CONTAINER 07=BURNING<br>04=COMMUNITY DRAIN 08=OTHER (SP.) …………………………………….		
62	Do you pay for disposing of household solid waste?<br>1=YES 2=NO		IF CODE 2, SKIP to 64
63	How much do you pay monthly?	Amount in GH C	
64	How do you dispose household liquid waste (waste water from bathing, preparation of food, cooking and other personal and domestic activities)?<br>1=SEPTIC TANK 2=COMMUNITY DRAIN 3=BACK OF HOUSE<br>4=INDISCRIMINATELY 5=OTHER (SPECIFY)……………………………		
65	What is the main source of cooking fuel for this household? *codes below*		
66	Does your household have……………?		

	1=YES 2=NO		1=YES 2=NO
CAR		WASHING MACHINE	
BICYCLE		TELEVISION	
BOAT/CANOE		RADIO	
TRUCK		TELEPHONE/Mobile Phone	
AN OUTBOARD MOTOR		CLOCK	
REFRIGERATOR		ELECTRIC/GAS STOVE	
FREEZER		SOFA	
GENERATOR		SEWING MACHINE	
IRON		ELECTRIC FAN	
COMPUTER		FISHING NET	

**Codes: Question 52**	**Codes: Question56**	**Codes: Ques.57**	**Codes: Q65**
01=OVERHEAD TANK<br>02=PLASTIC/STEEL CONTAINER WITH LID<br>03=PLASTIC/STEEL CONTAINER WITHOUT LID<br>04=EARTHEN WARE POT WITH LID<br>05=EARTHEN WARE POT WITHOUT LID<br>06=ALUMINIUM BUCKET WITH LID<br>07=ALUMINIUM BUCKET WITHOUT LID<br>08=BASIN(PLASTIC/ALUMINIUM/ENAMEL<br>09=SACHET<br>96=OTHER (SPECIFY)…………………………………	01=BOIL<br>02=ADD BLEACH, CHLORINE, OR ALLOY<br>03=STRAIN THROUGH A CLOTH<br>04=SOLAR DISINFECTION<br>05=LET IT STAND TO SETTLE<br>06=WATER TABLETS<br>07=ALUM<br>08=CAMPHOR<br>96=OTHER (SPECIFY)……………..<br>98= DON'T KNOW	01=NO FACILITY (BUSH/BEACH/FIELD)<br>02=WATER CLOSET (W.C)/FLUSH TOILET<br>03= KVIP<br>04= PIT LATRINE<br>05=BUCKET/PAN<br>06=PUBLIC TOILET (W.C,KVIP, PIT LATRINE,BUCKET/PAN)<br>96=OTHER (SPECIFY)…………………	01=NONE/NO COOKING<br>02= WOOD<br>03=GAS<br>04=ELECTRICITY<br>05=KEROSENE<br>06=CHARCOAL<br>07=CROP RESIDUE<br>08=SAW DUST<br>09=ANIMAL WASTE<br>96=OTHER (SPECIFY)……

67	How many rooms does this household occupy? (*count living, dining, bedrooms but not bathrooms ,toilet, kitchen& store room*)	
68	How many of the rooms are designed **<u>primarily</u>** for sleeping?	
69	How many household members sleep outside the designated sleeping rooms?<br>**CODE 00 IF NO HOUSEHOLD MEMBER SLEEPS OUTSIDE**	
70	Who owns this dwelling?<br>01=OWNED BY HH MEMBER 05=PRIVATE EMPLOYER<br>02=BEING PURCHASED (e.g., Mortgage) 06=OTHER PRIVATE AGENCY<br>03=RELATIVE NOT HH MEMBER 07=PUBLIC/GOVERNMENT OWNERSHIP<br>04=OTHER PRIVATE INDIVIDUAL 96=OTHER (SPECIFY)_____________	
71	What is the present holding/tenancy arrangement of this dwelling?<br>1=OWNING 2=RENTING 3=RENT FREE 4=PERCHING 5=SQUATTING 6=OTHER (SPECIFY)___________	

HOUSEHOLD OBSERVATION

72	What type of dwelling does this household occupy? ***Record observation***<br>01=SEPARATE HOUSE 05=SEVERAL HUTS/ BUILDING 09= ATTACHED TO SHOP<br>02=SEMI-DETACHED HOUSE 06=TENT 10= COMPOUND HOUSE<br>03=FLAT/APARTMENT 07=KIOSK 96=OTHER (SPECIFY)___________<br>04=ROOMS 08=CONTAINER	
73	What is the main material of the floor? ***Record observation***<br>01=EARTH/SAND 05=WOOD PLANKS 09=CERAMIC TILES/PORCELAIN<br>02=BURNT BRICKS 06=TERRAZO 10=VINYL TILES<br>03=CEMENT/CONCRETE GRANITE/MARBLE 07=WOOLEN CARPET 11=STONE<br>O4=WOOD 08=LINOLEUM/RUBBER CARPET	
74	What is the main material of the roof? **Record observation**<br>01=THATCH/PALM LEAF/SOD 06=ROOFING SHINGLES<br>02=RUSTIC MAT 07=ASBESTOS/SLATE ROOFING SHEETS<br>03=CARDBOARD 08=PALM/BAMBOO<br>04=METAL SHEETS 09=WOOD<br>05=BRICK TILES 10=CEMENT<br>96=OTHER (SPECIFY).....................	
75	What is the main material of the wall? **RECORD OBSERVATION**<br>01=CANE/PALM/TRUNKS 08=MUD BRICKS<br>02=BAMBOO WITH MUD 09=STONE WITH MUD<br>03= WOOD 10= PLYWOOD<br>04=CARDBOARD 11=BAMBOO<br>05=LANDCRETE 12=CEMENT BLOCKS/CONCRETE<br>06=BURNT BRICKS<br>07=METAL SHEETS/SLATE/ASBESTOS 96=OTHER (SPECIFY)...	
76	**INCOME EVALUATION QUESTION (IEQ)**<br>Taking into account your own situation with respect to the physical characteristics of your family and job you would call your net-income (including gifts from family and friends, and tips) per year	

	GHANA CEDIS	
MORE THAN WHAT YOU NEED (IF IT WERE ABOVE)		
JUST WHAT YOU NEED (IF IT WERE BETWEEN)		
BARELY WHAT YOU NEED (IF IT WERE BETWEEN)		
LESS THAN WHAT YOU NEED (IF IT WERE BETWEEN)		
MUCH LESS THAN WHAT YOU NEED (IF IT WERE BELOW)		

77	Who is the primary source of income for this household?<br>01=HEAD OF HOUSEHOLD 04= A DIFFERENT MEMBER OF THE HOUSEHOLD<br>02=PARTNER/SPOUSE 96=OTHER (SPECIFY).................................<br>03=BOTH SHARED EQUALLY (HEAD AND SPOUSE)	
78	How much can you rely on relatives outside of your household or friends for financial support if you need it**?** 1=A LOT 2=SOMETIMES 3=A LITTLE 4=NOT AT ALL	

NO	Question		RESPONSE		
79	Who manages sanitation resources in your community?<br><br>***Prove: Circle all that apply***		YES	NO	
		a. AMA/SUB-METRO	1	**2**	
		b. UNIT COMMITTEE	1	**2**	
		c. LOCAL WATER/SANITATION COMMITTEE	1	**2**	
		d. COMMUNITY DEVELOPMENT COMMITTEE	1	**2**	
		e. NGO	1	**2**	
		f. TRADITIONAL LEADER(S)	1	**2**	
		g. RELIGIOUS ORGANISATION(S)	1	**2**	
		h. PRIVATE INDIVIDUAL(S)	1	**2**	
		i. PRIVATE ORGANISATION	1	**2**	
		j. OTHER………………………..	1	**2**	
		k. DON'T KNOW	1	**2**	
80	What are the major environmental challenges that you face in this community		YES	NO	**IF NO TO ALL, SKIP TO Q84**
		a. FLOODING	1	**2**	
		b. POOR SANITATION	1	**2**	
		c. POLLUTION	1	**2**	
		d. HIGH TEMPERATURE (HEAT)	1	**2**	
		e. SEA LEVEL RISE	1	**2**	
		f. OTHER (SPECIFY)………..	1	**2**	
81	Which is the most challenging environmental issue mentioned in Ques.80 above? **Specify**_____________________________		☐		
82	What are the most common diseases in this community that are associated with these environmental challenges?		YES	NO	
		a. DIARRHOEA	1	**2**	
		b. MALARIA	1	**2**	
		c. CEREBRO-SPINAL MENINGITIS	1	**2**	
		d. SKIN RASH	1	**2**	
		e. COUGH/DIPHTHERIA	1	**2**	
		f. OTHER (SPECIFY)	1	**2**	
			1	**2**	
83	What is the most common disease mentioned in Ques.82 in this community that is associated with environmental challenges? **Specify**_____________________________		☐		
84	What is the distance from your house to the nearest standing water or open gutter? *Distance in meter*		☐☐☐☐		
85	When was the last time you or any member of your household had diarrhoea?<br>1=LESS THAN A WEEK AGO 2=A WEEK AGO 3=TWO WEEKS AGO<br>4=THREE WEEKS AGO 5=A MONTH AGO 6=MORE THAN A MONTH AGO 7=NEVER HAD DIARHHOEA		☐	**IF CODE 7, SKIP TO Q90**	
86	How many times did you or any member of your household have diarrhoea within the past month?		☐☐		
87	Did you or the member of your household seek advice or treatment for the diarrhoea (from any source)? 1=YES 2=NO		☐	**If code 2 SKIP to Q.90**	
88	Where did you first seek advice or treatment for the diarrhoea?<br>01=HOSPITAL/CLINIC 03= HERBAL MEDICINE (SELF PRESCRIPTION) 05=(SELF PRESCRIPTION)<br>02=PHARMACY/DRUG STORE 04=HERBAL MEDICINE 96=OTHER (SPECIFY)………………………		☐☐		
89	How many days after the diarrhoea began did you first seek advice or treatment?		☐☐		
90	The last time the youngest child (**under 5 years**) passed stool, what was done to dispose of the stool? **CHECK HH ROSTER IF THERE IS A CHILD UNDER 5 YEARS**<br>01=CHILD USED TOILET/LATRINE 06=LEFT IN THE OPEN<br>02=PUT/RINSED INTO TOILET OR LATRINE 07=NO CHILD UNDER 5 YEARS<br>03=PUT/RINSED INTO DRAIN OR DITCH 96=OTHER (SPECIFY)……………..<br>04=THROWN INTO GARBAGE 98=DON'T KNOW<br>05=BURIED		☐☐		

Household level conditions and adaptive capacity			
91	Have you noticed any change in climate (Rainfall and Temperature) for past 30 years?<br>1=YES 2= NO 8=DON'T KNOW	☐	**IF CODE 2 or 8, SKIP TO Q99**
92	What changes have you noticed? ***(circle all that apply)***		
93	How sure are you that the pattern of rainfall and temperature are changing?<br>1=Extremely sure 2=Sure 3=Somewhat sure 4=Not at all sure	☐	
94	How worried are you about the changing pattern of rainfall and temperature?<br>1=Very worried 2=Somewhat worried 3=Not worried 4=Not at all worried	☐	
95	What in your opinion causes these changes in rainfall and temperature?<br>***(circle all that apply)***		
96	Which of the above mentioned factors contribute **most** to changes in rainfall and temperature?<br>1=Emissions from cars and trucks 4=Toxic wastes 7=Cow rearing<br>2=Burning fuel for heat and electricity 5=Aerosol spray cans 8= Other (specify)<br>3=Deforestation 6=Volcanic eruptions	☐	
97	In your opinion, what is the effect of changing pattern of rainfall and temperature on the one's chances of getting malaria?<br>1=It promotes the breeding of mosquitoes<br>2=It increases one's chances of getting malaria<br>3=It does not affect incidence of malaria<br>4=Other, specify………………………………	☐	
98	In your opinion, what is the main course of action that can be taken to reduce the effect of changing pattern of rainfall and temperature on the incidence of malaria?<br>1=Use of mosquito net<br>2=Use mosquito coil/repellent<br>3=Clean our environment<br>4=Desilting clogged gutters<br>5=Other, specify ……………………….	☐	
99	Indicate which of the following reflects your household's capacity to prevent and treat malaria<br>1=My household has adequate capacity to prevent the incidence of malaria<br>2=My household has inadequate capacity to prevent the incidence of malaria<br>3=My household has adequate capacity to treat malaria<br>4= My household has inadequate capacity to treat malaria<br>5=My household has inadequate capacity to prevent and treat malaria<br>6= My household has adequate capacity to prevent and treat malaria	☐	

Q92:

	YES	NO
a. Getting more rainfall than before	1	2
b. Less rainfall than before	1	2
c. Rainfall becoming erratic/unpredictable	1	2
d. Increase in temperature	1	2
e. Decrease in temperature	1	2
f. Other (specify)………………………………………	1	2

Q95:

	YES	NO
a. Emissions from cars and trucks	1	2
b. Burning fuel for heat and electricity	1	2
c. Deforestation	1	2
d. Toxic wastes	1	2
e. Aerosol spray cans	1	2
f. Volcanic eruptions	1	2
g. Cow rearing	1	2
h. Other (specify)	1	2

No.	Question	Code	
100	The last time you or any member of your household was diagnosed of malaria, were you made to undergo a laboratory test for confirmation<br>1 = YES 2 = NO	☐	
101	If household members do not seek treatment from health facility, what reason do they have for not seeking treatment from health facility<br>1= I believe the herbs are effective<br>2= Lack of money<br>3= Delays/long queue in the hospital<br>4= Treatment in the facility not effective<br>5= Household sought treatment<br>6= Other (specify)……………………………………………………………….	☐	
102	Considering your household's condition as well as the prevailing conditions in your community, how would you rate your household risk or vulnerability to the incidence of malaria on a scale of 0-3?<br>0=No Risk<br>1=Small<br>2=Moderate<br>3=Great	☐	
103	During the last rainy season (May to July) did your community experience any flooding? 1 = YES 2 = NO	☐	**IF CODE 2 SKIP TO Q108**
104	How many times did you experience it within the season?	☐☐	
105	If flooding occurred in your community during the last season, how many days did it take for the area to completely dry up?	☐☐	
106	If flooding occurred in your community during the last season, how would you rate it:<br>1= MILD<br>2= MODERATE<br>3=SEVERE	☐	
107	How often is your house affected by flooding in a year?<br>1=Once a year 2=Two or more times a year 3=Not affected at all	☐	
108	What in your opinion causes malaria? *(tick those mentioned)*<br>1. Bites from infected mosquitoes ☐<br>2. Dirty environment ☐<br>3. Too much exposure to sunlight ☐<br>4. Eating oil rice ☐<br>5. Bites from mosquitoes ☐<br>6. Other (specify) ________________ ☐		
109	On the average how much do you spend on the treatment of each episode of malaria?	Amount in GHC	
110	The last time you or any member of your household was sick of malaria, how much did you or the person spend on malaria <u>**medication**</u>?	Amount in GHC	
111	Are the windows in the house covered with mosquito-proof screens/net?<br>1 = YES 2 = NO	☐	
112	On average, how much does your household spend on the following each month?<br>1=MOSQUITO COILS<br>2=MOSQUITO SPRAY<br>3=MOSQUITO REPELLING CREAM<br>4=NO AMOUNT SPENT	Amount in GHC	
113	What is the total amount of money you and the members of your household spend on health related issues each month, on the average?	Amount in GHC	

114	How often do you receive health related information or assistance in this community?<br>(from any source) 1=At least once a year<br>2=More than once a year 3=Not at all	☐	**IF CODE 3 SKIP TO Q116**
115	What is the **main** source of the information?<br>1=TV 5=MOSQUES/CHURCHES 9=WORK PLACE<br>2=RADIO 6=SCHOOLS/TEACHES 10=DRAMA /PERFORMANCE<br>3=NEWSPAPER 7=COMMUNITY MEETING 11=OTHER (SPECIFY_______<br>4=PAMPLETS/POSTERS 8=FRIENDS/ RELATIVES	☐	

	Is there any institution you turn to for help when you have health related problems in this community? ***(Tick all that apply)*** **IF NONE, SKIP TO Q121**	Mention the kind of support received. *Codes below**	Specify amount in monetary terms or specify type of kind	Have you received any support from organisation in the past one year? **1=Yes 2=No**	How would you rate the support received? *Codes below***
	**(116)**	**(117)**	**(118)**	**(119)**	**(120)**
Community based organization					
Private organization					
Government agency					
Other(specify)					

**Codes for Q117***	**CodesQ120****
1=Monetary	1=Able to meet all the need of household at the time
2=In kind	2=Somewhat able to meet the need of household?
3=Moral/psychological	3=Not helpful at all

HOUSEHOLD AND FAMILY SUPPORT NETWORKS AND TRANSFERS

The next questions are about your family and friends, specifically those not living with you in this household. Families and friends sometimes help one another in a variety of ways, and each type of help or support can be important. Part of our survey involves finding out how they do that. We would now like to ask some questions about your family and friends who do not live with you, and the different ways in which you help or support each other. The next questions are about help received by your household in the last 12 months.

121	Has any member of the household **received** any financial credit/loan within the past one year? 1 = YES 2 = NO	☐	
122	In the last 12 months, has anyone in the household **received** any financial or in-kind support from your family (children, siblings or parents), relatives (other kin) and friends who do not live with you?<br>1=YES 2=NO 8=DON'T KNOW	☐	**IF CODE 2 OR 8 SKIP TO Q126**
123	What type of assistance did your household receive?<br>1=MONEY ONLY 2=KIND ONLY (Specify)……………………………………<br>3=BOTH MONEY AND KIND (specify)………………………………………………………	☐	
124.	What are the purposes of the support?<br>***(Tick all that apply)***<br>a. General and household upkeep<br>b. Education<br>c. Medical care<br>d. Business and work related<br>e. Social events<br>f. Other (specify)………………………………………………………………………………	☐<br>☐<br>☐<br>☐<br>☐<br>☐	

125.	About how much would this assistance amount to over the last 12 months in Ghana Cedi (GH¢) ***(if in kind impute and estimate the value)***	GH¢ 0 0 0 0 0 0	
126.	In the last 12 months, has your household **provided** any financial aid or in-kind support to any of your children, grandchildren and/or other family (and those of your spouse) who do not live in this household?<br>01=YES 02=NO 8=DON'T KNOW	☐	**IF CODE 2 OR 8 SKIP TO Q130**
127.	What type of assistance did your household provide?<br>1=MONEY ONLY 3=BOTH MONEY AND KIND<br>2=KIND ONLY (Specify)…………………………………………………………………	☐	
128.	What are the purposes of the support provide?<br>*(Tick all that apply)*<br>a. General and household upkeep<br>b. Education<br>c. Medical care<br>d. Business and work related<br>e. Social events<br>f. Other	☐ ☐ ☐ ☐ ☐ ☐	
129.	Can you give an approximate total amount for this for the last 12 months in Ghana Cedis (GH¢) ***(if in kind impute and estimate the value)***	GH¢ 0 0 0 0 0 0	
130.	During the past year, did you or someone in your household provide help to a relative or friend (adult or child), because this person has a long-term physical, or mental illness, or disability, or is getting old and weak?<br>1=YES, physical illness 4= YES, GETTING OLD AND WEAK<br>2=YES, mental illness 5=NO<br>3= YES, DISABILITY	☐	

FOOD SITUATION AND EXPENDITURE

131. Was there a day in the **previous 30 days** when you or any member of the household did not have enough food to eat?
0=No 1=Between 1-5 days 2=Between 6-10 days 3=More than 10 days ☐

132. Please indicate whether your household bought cooked food (breakfast, lunch and dinner) from street vendors for each of the days within the past week.
(Tick all that apply)

DAY	BREAKFAST	LUNCH	DINNER
Monday			
Tuesday			
Wednesday			
Thursday			
Friday			
Saturday			
Sunday			

133. Please indicate the total expenditure your household made on cooked food (breakfast, lunch and dinner) from street vendors within the past week.

BREAKFAST	LUNCH	DINNER
Amount in GH C	Amount in GH C	Amount in GH C

END TIME FOR INTERVIEW **HOURS MINS**

Do you have any question or comments for me?

………
………
………
………
………
………
………
………
………
………
………
………
