# Supplementary material for: Household food sources and diarrhoea incidence in poor urban communities, Accra Ghana
Source: PLoS One. 2021 Jan 28;16(1):e0245466. doi: 10.1371/journal.pone.0245466 (PMC7842991; doi:10.1371/journal.pone.0245466)
Supplement: S1 File — (ZIP) [file pone.0245466.s001.zip › questionnaires and dataset - diarrhoea and food sources/Wave 3 - RIPS INDIVIDUAL QUESTIONNAIRE.pdf]

REGIONAL INSTITUTE FOR POPULATION STUDIES
UNIVERSITY OF GHANA

POPULATION TRAINING AND RESEARCH CAPACITY FOR DEVELOPMENT (POPTRCD)

URBAN HEALTH AND POVERTY PROJECT

INDIVIDUAL QUESTIONNAIRE

START TIME FOR INTERVIEW **HOURS MINS**

IDENTIFICATION

LOCALITY NAME* ______

E.A. BASE ______

NAME OF HOUSEHOLD HEAD ______

E.A. /EDL NUMBER ……

STRUCTURAL NUMBER……

HOUSEHOLD NUMBER ……

GREATER ACCRA…… 0 3

ROUND…… 0 3

* **CODES FOR LOCALITY NAME:** 1=AGBOGBLOSHIE 2=JAMES TOWN 3=USSHER TOWN

INTERVIEWER VISITS

	1	2	3	FINAL VISIT
DATE	______	______	______	DAY<br>MONTH<br>YEAR 2 0 1 3
INTERVIEWER'S NAME<br>RESULT*	______<br>______	______<br>______	______<br>______	INT. CODE<br>RESULT
Next visit: Date<br>Time	______<br>______	______<br>______		TOTAL NO. OF VISITS

NAME AND LINE NO. OF RESP. FROM HH QUEST.______

RESPONDENT INTERVIEWED IN ROUND 2 1=YES 2=NO

***RESULT CODES:**
1 COMPLETED
2 PARTLY COMPLETED
3 NO HOUSEHOLD MEMBER AT HOME OR NO COMPETENT RESPONDENT AT HOME AT THE TIME OF VISIT
4 ENTIRE HOUSEHOLD ABSENT FOR EXTENDED PERIOD OF TIME
5 POSTPONED
6 REFUSED
7 DWELLING VACANT OR ADDRESS NOT A DWELLING
8 DWELLING DESTROYED
9 DWELLING NOT FOUND
10 OTHER (SPECIFY) ______

LANGUAGE

LANGUAGE OF QUESTIONNAIRE: **ENGLISH** 1

LANGUAGE OF INTERVIEW** ______

NATIVE LANGUAGE OF RESPONDENT** ______

WAS TRANSLATOR USED? (YES=1, NO=2) ……

****LANGUAGE CODES**:
1 ENGLISH 2 AKAN 3 GA 4 EWE 5 DAGBANI 6 HAUSA
7 OTHER (SPECIFY) ______

SUPERVISOR	FIELD EDITOR	KEYED BY
NAME ______<br>DATE ______	NAME ______<br>DATE ______	

SECTION 1: RESPONDENT BACKGROUND AND MOBILITY			
Now, I would like to ask you some background information about yourself.			
**Q NO.**	**QUESTION**	**RESPONSE**	**SKIP**
100.	Sex of respondent<br>1=FEMALE 2=MALE	☐	
101.	In what month and year were you born?<br>**(CODE 98 FOR MONTH AND/ 9998 FOR YEAR IF DON'T KNOW MONTH AND/YEAR)**	**MONTH** ☐☐<br>**YEAR** ☐☐☐☐	
102.	How old were you on your last birthday?<br>**COMPARE AND CORRECT 101 AND/OR 102 IF INCONSISTENT**	☐☐	
103.	Have you ever attended school?<br>1=YES 2=NO	☐	**IF CODE 2 SKIP Q109**
104.	What is the highest level of education you have attained?<br>0==PRE-SCHOOL 1=PRIMARY 2=MIDDLE/JHS 3= SECONDARY/SHS 4=HIGHER	☐	
105.	What is the highest grade you have completed at that level?	**GRADE** ☐☐	
106.	Are you currently attending school?<br>1=YES 2=NO	☐	

107.	Who contributes/contributed to your school-related expenses?<br>**PROBE: WHO ELSE?**	**YES**	**NO**
	a. FATHER	1	2
	b. MOTHER	1	2
	c. OTHER RELATIVES	1	2
	d. FRIEND	1	2
	e. SCHOOL	1	2
	f. TEACHER	1	2
	g. NON GOVERNMENTAL ORGANIZATION	1	2
	h. RELIGIOUS GROUP	1	2
	i. SELF	1	2
	j. OTHER (SPECIFY)..	1	2

CHECK Q106: IF NOT CURRENTLY ATTENDING SCHOOL GO TO Q108 ☐
IF CURRENTLY ATTENDING SCHOOL SKIP TO Q110 ☐

108.	What were the reasons why you stopped attending school?<br>**PROBE: ANY OTHER? (TICK MOST IMPORTANT √)**	**YES**	**NO**	**MOST IMPORTANT**	
	a. GOT PREGNANT	1	2		**SKIP TO Q110**
	b. GOT MARRIED	1	2		
	c. TO CARE OF SIBLINGS	1	2		
	d. FAMILY NEEDED HELP ON FARM OR IN BUSINESS	1	2		
	e. COULD NOT PAY SCHOOL FEES	1	2		
	f. NEEDED TO EARN MONEY	1	2		
	g. COMPLETED AT THAT LEVEL	1	2		
	h. HAD ENOUGH SCHOOL	1	2		
	i. DID NOT PASS EXAM	1	2		
	j. DID NOT LIKE SCHOOL	1	2		
	k. SCHOOL NOT ACCESSIBLE /TOO FAR	1	2		
	l. PHYSICALLY/MENTALLY CHALLENGED	1	2		
	m. POOR SCHOOL QUALITY	1	2		
	n. EXPELLED	1	2		
	o. FAMILY SEES NO ECONOMIC BENEFIT	1	2		
	p. OTHER (SPECIFY)	1	2		
	q. DON'T KNOW	1	2		

109. What were the reasons you never attended school?
(PROBE: ANY OTHER?) (TICK MOST IMPORTANT √)

		YES	NO	MOST IMPORTANT
a.	TO CARE FOR SIBLINGS	1	2	
b.	TOO MANY DOMESTIC/FAMILY RESPONSIBILITIES	1	2	
c.	FAMILY COULD NOT PAY SCHOOL FEES	1	2	
d.	DID NOT LIKE SCHOOL	1	2	
e.	SCHOOL TOO FAR/NOT ACCESSIBLE	1	2	
f.	PHYSICALLY/MENTALLY DISABLED	1	2	
g.	FAMILY SEES NO BENEFIT	1	2	
h.	OTHER (SPECIFY)…………………………………………	1	2	
i.	DON'T KNOW	1	2	

110. Where were you born?

Specify name of locality/Country ___________________________
and Region.

***REGION** [ ][ ]

***CODES FOR REGION**
01=WESTERN 02=CENTRAL 03=GREATER ACCRA 04=VOLTA
05=EASTERN 06=ASHANTI 07=BRONG AHAFO 08=NORTHERN
09=UPPER EAST 10=UPPER WEST 11=OTHER

111. How many years have you lived in this community in total?
(CODE 99 IF SINCE BIRTH, CODE 15 FOR LESS THAN ONE MONTH IN MONTH AND CODE 98 IF DON'T KNOW)

MONTHS [ ][ ]
YEARS [ ][ ]
SINCE BIRTH [ ][ ]

IF SINCE BIRTH SKIP TO Q121

112. Just before you moved to this community, where did you live?

Specify community/Country_______________________________ and record region in which the community is located?

***REGION** [ ][ ]

***CODES FOR REGION**
01=WESTERN 02=CENTRAL 03=GREATER ACCRA 04=VOLTA
05=EASTERN 06=ASHANTI 07=BRONG AHAFO 08=NORTHERN
09=UPPER EAST 10=UPPER WEST 11=OTHER

113. Where did you mostly live during the first 15 years of your life?

Specify community/Country_________________________________ and record region in which the community is located?

***REGION** [ ][ ]

***CODES FOR REGION**
01=WESTERN 02=CENTRAL 03=GREATER ACCRA 04=VOLTA
05=EASTERN 06=ASHANTI 07=BRONG AHAFO 08=NORTHERN
09=UPPER EAST 10=UPPER WEST 11=OTHER

114. Why did you move to this community?

01=NO REASON 2=TO LOOK FOR A JOB 3=TO LEARN A TRADE 4=TO ESTABLISH HH 5=TO JOIN PARTNER 6=ACCOMODATION PROBLEM 7=CLOSER TO WORK 8=PROBLEMS WITH FAMILY 9=FAMILY RE-UNION 10=OTHER (SPECIFY)………………………………………………

[ ][ ]

115. Do you ever visit your last community?

1 = YES 2 = NO

[ ]

IF CODE 2 SKIP TO Q119

116. How many times in the last 12 months do you visit that community?

TIMES PER YEAR [ ][ ][ ]

117. What is the main reason why you visit that community?
1=VISIT FAMILY 2=VISIT FRIENDS 3=ATTEND FUNCTIONS
4=WORK/TRAINING/SCHOOL 5=UTILISE SERVICE
6=OTHER (SPECIFY)…………………………………………………………………

[ ]

118. Which do you consider your primary residence?

1= CURRENT COMMUNITY 2=PLACE OF BIRTH
3=BOTH EQUALLY (1 AND 2) 4=A DIFFERENT COMMUNITY

[ ]

CHECK HH COL 5: USUAL RESIDENT GO TO Q120 ☐			
119.	At the time you moved here, did you<br>1=MOVE TO JOIN EXISTING HH 2=MOVE WITH OTHERS TO START HH<br>3=MOVE ALONE 4=WHOLE HOUSEHOLD MOVED<br>5=OTHER (SPECIFY)___________________________	☐	

120.	Who made the decision for you to move to this community?<br>**PROBE: ANY OTHER?**	YES	NO
	a. SELF	1	2
	b. SPOUSE	1	2
	c. SELF AND SPOUSE	1	2
	d. PARENT(S)	1	2
	e. CHILD/CHILDREN	1	2
	f. OTHER RELATIVES	1	2
	g. EMPLOYER	1	2
	h. GOV'T RESETTLEMENT	1	2
	i. OTHER (SPECIFY) ___________________________	1	2
	j. DON'T KNOW	1	2

121.	Do you plan to move out of this ***structure*** in the future?<br>1=YES, JUST MYSELF 3=NOT CERTAIN<br>2=YES, WHOLE HOUSEHOLD 4=NO 5=OTHER SPECIFY..	☐	**IF CODE 3 SKIP TO Q126**<br>**IF CODE 4 SKIP TO Q125**
122.	Where do you plan to go?<br>01=WITHIN THE SAME COMMUNITY<br>02=PART OF SLUM IN ACCRA (SPECIFY) ....<br>03=PART OF NON SLUM IN ACCRA (SPECIFY) ....<br>04=ANOTHER TOWN (SPECIFY) ....<br>05=A VILLAGE (SPECIFY) ....<br>06=BOARDING SCHOOL<br>96=OTHER (SPECIFY)...	☐☐	
123.	How soon are you planning to move?<br>**(IF LESS THAN A YEAR, ENTER MONTHS AND CODE 00 FOR YEARS. ENTER 98 FOR BOTH MONTHS AND YEARS IF DON'T KNOW/UNSURE) IF LESS THAN A MONTH CODE 15 IN MONTHS**	**MONTHS** ☐☐<br>**YEARS** ☐☐	

124.	Why do you want to move out? **PROBE: ANY OTHERS? RECORD ALL RESPONSES (TICK MOST IMPORTANT √)**	YES	NO	MOST IMPORTANT	
	a. JOB ELSEWHERE	1	2		**SKIP TO Q126**
	b. NO WORK AVAILABLE HERE	1	2		
	c. RENT TOO HIGH	1	2		
	d. MARRIAGE	1	2		
	e. WHOLE FAMILY MOVING	1	2		
	f. FAMILY PROBLEMS	1	2		
	g. TO ESTABLISH OWN RESIDENCE	1	2		
	h. WAS HERE TEMPORARILY	1	2		
	i. CAN AFFORD BETTER HOUSE	1	2		
	j. TOO MUCH CRIME/DRUGS	1	2		
	k. RETIRED/LOST JOB	1	2		
	l. OTHER (SPECIFY)..	1	2		

125.	What are the main reasons for not wanting to move?<br>**PROBE: ANY OTHERS? REECORD ALL RESPONSES (TICK MOST IMPORTANT √)**	YES	NO	MOST IMPORTANT
	a. CAN'T AFFORD TO MOVE	1	2	
	b. HAPPY WITH JOB	1	2	
	c. FAMILY LIVES HERE	1	2	
	d. OWN PROPERTY HERE	1	2	
	e. HAVE NOWHERE ELSE TO GO	1	2	
	f. TOO OLD TO MOVE	1	2	
	g. RENT IS CHEAPER	1	2	
	h. JOB RELATED REASONS	1	2	
	i. SECURITY IS GOOD	1	2	
	j. OTHER (SPECIFY)..	1	2	

126.	What is your religion?<br>01=NO RELIGION 02=CATHOLIC 03=PROTESTANTS<br>04=PENTECOSTAL/CHARISMATIC 05=OTHER CHRISTIAN<br>06=ISLAM 07=TRADITIONAL/SPIRITUALIST 08=EASTERN RELIGIONS<br>96=OTHER (SPECIFY) …………………………………………		
127.	How often do you pray?<br>1=AT LEAST ONCE A DAY 2=AT LEAST ONCE A WEEK 3=AT LEAST ONCE A MONTH<br>4=NEVER		
128.	How important is religion to you?<br>1=VERY IMPORTANT 2= IMPORTANT 3= INDIFFERENT<br>4=NOT IMPORTANT 5=NOT IMPORTANT AT ALL		
129.	In the past one (1) month, how often did you attend religious services?<br>**(CODE 98 IF DON'T KNOW)**		
130.	What is your ethnic group?<br>01=AKAN 02=GA-DANGME 03=EWE 04=GUAN 05=GRUMA 06=MOLE-DAGBANI<br>07=GRUSSI 08=MANDE 96=OTHER (SPECIFY)………………………………		
131.	Is your biological mother alive?<br>1=YES 2=NO 8=DON'T KNOW		**IF CODE 1, 8 SKIP TO Q133**
132.	How old were you when your mother died?<br>**AGE IN COMPLETED YEARS IF DON'T KNOW CODE 98**		
133.	Is your biological father alive?<br>1=YES 2=NO 8=DON'T KNOW		**IF CODE 1, 8 SKIP TO Q135**
134.	How old were you when your father died?<br>**AGE IN COMPLETED YEARS IF DON'T KNOW CODE 98**		
135.	Who are you living with?<br>01= ALONE 02=PARENT(S) 03= SPOUSE/PARTNER<br>04= CHILD(REN) 05=SPOUSE/PARTNER AND CHILDREN 06=FRIEND<br>07=SPOUSE/PARTNER &/ CHILD(REN) & OTHERS 08=SIBLINGS 09=GRANDPARENT(S)<br>10=OTHER (SPECIFY)…………………………………………………………………		
**CHECK Q131 AND Q133: BOTH PARENTS ALIVE GO TO Q 136**		**ONE OR BOTH DEAD PARENTS DEAD SKIP TO Q137**	
136.	Are your mother and father currently married to each other?<br>1=YES, CURRENTLY MARRIED 2=NO, NOT CURRENTLY MARRIED<br>3=NEVER MARRIED TO EACH OTHER 8=DON'T KNOW		**IF CODE 1, 3, SKIP TO Q138**
137.	Were your parents ever married to each other?<br>1=YES 2=NO 8=DON'T KNOW		
138.	What is (was) the highest level of education your mother completed?<br>0=NO EDUCATION 1=PRE-SCHOOL 2=PRIMARY 3=MIDDLE/JHS<br>4=SECONDARY/SHS 5=HIGHER 8=DON'T KNOW		
139.	What is (was) the highest level of education your father completed?<br>0=NO EDUCATION 1=PRE-SCHOOL 2=PRIMARY 3=MIDDLE/JHS<br>4=SECONDARY/SHS 5=HIGHER 8=DON'T KNOW		
140.	What kind of work did/does your father do?<br>…………………………………………………………………………………………<br>01=NO OCCUPATION 02=PROFESSIONAL/TECHNICAL 03=MANAGEMENT<br>04=CLERICAL 05=SALES 06=AGRICULTURE- SELF EMPLOYED 07=AGRICULTURE<br>08=HOUSEHOLD AND DOMESTIC 09=SERVICE<br>10=SKILLED MANUAL 11=UNSKILLED MANUAL<br>12=OTHER (SPECIFY)………………………………………………<br>98=DON'T KNOW		
141.	What kind of work did/does your mother do?<br>…………………………………………………………………………………………<br>01=NO OCCUPATION 02=PROFESSIONAL/TECHNICAL 03=MANAGEMENT<br>04=CLERICAL 05=SALES 06=AGRICULTURE- SELF EMPLOYED 07=AGRICULTURE<br>08=HOUSEHOLD AND DOMESTIC 09=SERVICE<br>10=SKILLED MANUAL 11=UNSKILLED MANUAL<br>12=OTHER (SPECIFY)………………………………………………<br>98=DON'T KNOW		

142.	Are you currently working?<br>1=YES 2=NO		IF CODE 1 SKIP TO Q146
143.	As you know, some people take up jobs for which they are paid in cash or kind. Others sell things, have small business or work on the family farm or in the family business, others are in school. Are you currently doing any of these things or doing any other work?<br>1=YES 2=NO		IF CODE 1, SKIP TO Q147
144.	What have you been doing for most of the time over the last 12 months?<br>1=GOING TO SCHOOL /STUDYING 2=LOOKING FOR WORK 3=INACTIVE<br>4=COULD NOT WORK /HANDICAPPED<br>8=OTHER (SPECIFY)…………………………………………………………………………………		IF CODE 1 SKIP TO SECTION 2
145.	Have you done any work in the last 12 months?<br>1=YES 2=NO		IF CODE 1 SKIP TO Q147. IF CODE 2, SKIP TO SECTION 2
146.	During the last 12 months, how many months did you work?	NO. OF MONTHS	SKIP TO Q148
147.	How long did you do/have you been doing this particular work in the last 12 months? **IF MULTIPLE JOBS ASK ABOUT THE MAIN JOB**	NO. OF MONTHS	
148.	How many days do/did you spend on this work each week?	NO. OF DAYS	
149.	Do/Did you do this work for a member of your family, for someone else or are you self-employed?<br>1=FOR FAMILY MEMBER 2=FOR SOMEONE ELSE (govt &private)<br>3=SELF-EMPLOYED		IF CODE 1, 2 SKIP TO Q151
150.	How many paid employees do you have?<br>**IF NOT APPLICABLE CODE 99**	NO. OF EMPLOYEES	
151.	Do you usually work at home or away from home?<br>1=HOME 2=AWAY		
152.	How many work days were you away from work in the past month due to your own illness or injury, or that of other family members?<br>**NOT WORKED IN PAST MONTH, CODE 95**	NO. OF DAYS	
153.	Do you usually work throughout the year, or do you work seasonally, or only once in a while?<br>1=THROUGHOUT THE YEAR 2=SEASONALLY/PART OF THE YEAR 3=ONCE IN A WHILE		
154.	Does/Did your employment require you to work at night?<br>1=ALWAYS 2=SOMETIMES 3=NEVER 4=NOT WORKING		
155.	What is your occupation, that is, what kind of work do you mainly do?<br>**NAME OF OCCUPATION**--<br>01=NO OCCUPATION 02=PROFESSIONAL/TECHNICAL 03=MANAGERIAL<br>04=CLERICAL 05=SALES 06=AGRICULTURE- SELF EMPLOYED 07=AGRICULTURE<br>08=HOUSEHOLD AND DOMESTIC 09=SERVICE<br>10=SKILLED MANUAL 11=UNSKILLED MANUAL<br>12=OTHER (SPECIFY)………………………………………………<br>98=DON'T KNOW		IF CODE 01 SKIP TO SECTION 2
156.	Do you think the work you are doing now/did fit your skill/qualification?<br>1=YES 2=NO 3=REQUIRE NO SKILLS		IF CODE 1 SKIP TO Q158
157.	Which occupation best fits your skill/qualification?<br>………………………………………………………………………………… **HAVE NO SKILLS, CODE 94**		
158.	Are you paid or do you earn cash or in kind for this work or are you not paid at all?<br>1=CASH ONLY 2=CASH AND KIND 3=IN KIND ONLY 4=NOT PAID		IF CODE 3, 4 GO TO NEXT SECTION

159.	How much do you earn for this work per month?<br>RECORD AMOUNT (GH¢) →	AMOUNT (GH¢)
160	Who mainly decides how the money you earn will be used?<br>1=SELF 2=SPOUSE/PARTNER<br>3=SELF AND PARTNER JOINTLY 4=SOMEONE ELSE<br>5=JOINTLY WITH SOMEONE ELSE 6=PARENT(S)	
161	On average, how much of your household's expenditures do your earnings pay for:<br>1=ALMOST NONE 2=LESS THAN HALF 3=ABOUT HALF<br>4=MORE THAN HALF 5=ALL	

SECTION 2: SHOULD BE ADMINISTERED TO MALE AND FEMALE RESPONDENTS.

SECTION 2: COMMUNITY AND ENVIRONMEMT

Now I am going to ask you questions about your community and the environment

201. When I say the word "community", do you think of an area:

1=WITHIN A FEW MINUTES' WALK FROM YOUR HOME?
2=WITHIN A 10-MINUTE WALK FROM YOUR HOME?
3=MORE THAN 10-MINUTE WALK FROM YOUR HOME?

202. On a scale of 1 (low) to 5 (high), how much of a problem are the following in your community:

	SCALE				
a. DETERIORATION AND POOR SANITATION	1	2	3	4	5
b. POVERTY	1	2	3	4	5
c. CRIME	1	2	3	4	5
d. LACK OF EMPLOYMENT	1	2	3	4	5
e. DRUG SELLING OR USE	1	2	3	4	5
f. EXCHANGING SEX FOR MONEY	1	2	3	4	5
g. UNSAFE SEXUAL PRACTICES	1	2	3	4	5
h. INSUFFICIENT HEALTH CARE	1	2	3	4	5
i. GROUPS OF TEENAGERS OR ADULTS CAUSING TROUBLE	1	2	3	4	5
j. TRUST IN LOCAL POLICE	1	2	3	4	5
k. DIFFERENT SOCIAL GROUP WHO DO NOT GET ALONG WITH EACH OTHER	1	2	3	4	5
l. THE POLICE NOT PATROLLING THE AREA OR RESPONDING TO CALLS IN THE AREA	1	2	3	4	5
m. OTHER (SPECIFY)...	1	2	3	4	5

203. For each statement, tell me whether you:

1=STRONGLY DISAGREE 2=DISAGREE 3= AGREE 4=STRONGLY AGREE

	SCALE			
a. THIS IS A CLOSE-KNIT COMMUNITY	1	2	3	4
b. PEOPLE IN THIS COMMUNITY ARE WILLING TO HELP EACH OTHER	1	2	3	4
c. PEOPLE IN THIS COMMUNITY CAN BE TRUSTED	1	2	3	4
d. PEOPLE IN THIS COMMUNITY WATCH OUT FOR EACH OTHER	1	2	3	4
e. PEOPLE IN THIS COMMUNITY WOULD WORK TOGETHER IF THERE WAS A SERIOUS PROBLEM	1	2	3	4
f. PEOPLE IN THIS COMMUNITY LOOK OUT MAINLY FOR THE WELFARE OF THEIR FAMILIES AND THEY ARE NOT MUCH CONCERNED WITH COMMUNITY WELFARE.	1	2	3	4

204. How many out of ten adult neighbours do you know by name?

205. About how many minutes per week would you say you spend talking to neighbours?

NO. OF MINS.

206. How likely are you to **ask** for help from a neighbour if you needed it

1=VERY LIKELY 2= SOMEWHAT LIKELY 3= NOT VERY LIKELY 4= NEVER

	SCALE			
a. TO BORROW A SMALL AMOUNT OF MONEY	1	2	3	4
b. A SMALL AMOUNT OF FOOD	1	2	3	4
c. SOMEWHERE TO SPEND THE NIGHT	1	2	3	4
d. MEDICINE OR MEDICAL CARE	1	2	3	4
e. TO TALK ABOUT SOMETHING WORRYING YOU	1	2	3	4

207. How likely do you think you would be able to **receive** help from a neighbour if you asked:

1=VERY LIKELY 2= SOMEWHAT LIKELY 3= NOT VERY LIKELY 4= NEVER

	SCALE			
a. TO BORROW A SMALL AMOUNT OF MONEY	1	2	3	4
b. A SMALL AMOUNT OF FOOD	1	2	3	4
c. SOMEWHERE TO SPEND THE NIGHT	1	2	3	4
d. MEDICINE OR MEDICAL CARE	1	2	3	4
e. TO TALK ABOUT SOMETHING WORRYING YOU	1	2	3	4

208. How likely are you to **help** a neighbour who needed:

1=VERY LIKELY 2= SOMEWHAT LIKELY 3= NOT VERY LIKELY 4= NEVER

	SCALE			
a. TO BORROW A SMALL AMOUNT OF MONEY	1	2	3	4
b. A SMALL AMOUNT OF FOOD	1	2	3	4
c. SOMEWHERE TO SPEND THE NIGHT	1	2	3	4
d. MEDICINE OR MEDICAL CARE	1	2	3	4
e. TO TALK ABOUT SOMETHING WORRYING THEM	1	2	3	4

209.	How likely are you to get help from a friend or relative **outside** the community if you needed:<br>**1=VERY LIKELY 2= SOMEWHAT LIKELY 3= NOT VERY LIKELY 4= NEVER**	**SCALE**					
	a. TO BORROW A SMALL AMOUNT OF MONEY	1	2	3	4		
	b. A SMALL AMOUNT OF FOOD	1	2	3	4		
	c. SOMEWHERE TO SPEND THE NIGHT	1	2	3	4		
	d. MEDICINE OR MEDICAL CARE	1	2	3	4		
	e. TO TALK ABOUT SOMETHING WORRYING YOU	1	2	3	4		

210.	To what extent do you receive support from other members of this community<br>1. I receive more than I give out<br>2. I receive less than I give out<br>3. I receive just about the same I give out<br>4. I do not receive any support	☐	
211.	Read each question and enter the most appropriate response. Enter only **ONE** of the following answers for **EACH RESPONSE**: HAVE YOU…<br>**1=NEVER 2=JUST ONCE OR TWICE 3=SEVERAL TIMES**<br>**4=ALWAYS 8=DON'T KNOW**		
	a. FEARED CRIME IN YOUR OWN HOME?	☐	
	b. HAD YOUR HOME BEEN BROKEN INTO WITH SOMETHING STOLEN?	☐	
	c. BEEN PHYSICALLY ATTACKED?	☐	
	d. EXPERIENCED DOMESTIC VIOLENCE?	☐	
	e. HAD SOMETHING STOLEN FROM YOU?	☐	
	f. BEEN RAPED?	☐	
	g. FEARED WALKING IN YOUR OWN NEIGHBOURHOOD?	☐	
	h. BEEN A VICTIM OF WITCHCRAFT	☐	
	i. RECEIVED THREATS ON YOUR LIFE	☐	
	j. RECEIVED WARNINGS OF PLANNED ARMED ROBBERY OF YOUR HOME	☐	
212.	How many friends would you say you have close relationship(s) with in this community?	☐☐	

213.	Are you currently a member of any association or group in this community? 1=YES 2=NO	☐	**IF CODE 2 SKIP TO Q219**
214.	What type of group do you belong to?<br>(Probe: What else?)	**YES**	**NO**
	a. RELIGIOUS (CHURCH, ISLAMIC ETC...)	1	2
	b. MOTHER –TO –MOTHER SUPPORT/MOTHERS' CLUB OR FATHER'S CLUB	1	2
	c. HOME TOWN ASSSOCIATION	1	2
	d. CREDIT ASSOCIATION	1	2
	e. MARKET GROUP/TRADERS ASSOCIATION	1	2
	f. BUSINESS COOPERATIVE	1	2
	g. RECREATIONAL ASSOCIATION (FOOTBALL, BOXING, KEEP FIT ETC)	1	2
	h. OTHER (SPECIFY)…………………………………………………………………	1	2
215.	Did you contribute money or goods to this group(s)? 1=YES 2=NO	☐	**IF CODE IS 2 SKIP Q217**
216.	How much money or goods did you contribute to this group(s) in the past 12 months?		

	**Specify amount / type goods**								
Money		Ghana cedis					Pesewas		
	GH								
Good									
Good									

217.	How many days of work did you give to this group(s) in the past 12 months?<br>**IF NONE CODE=000**		
218.	What is the main benefit from joining this group(s)?<br>1. Improves my current livelihood or access to services<br>2. Important in times of emergency/need<br>3. Benefits the community<br>4. Enjoyment/recreation<br>5. Spiritual/social status/self-esteem		
219.	Consider the economic status of the residents in your community, where the people at the top (5) have the highest economic standing in the community and people at the bottom (1) have the lowest standing. Which number (1 to 5) best represents where you stand at this time in your life, relative to other people in your **current** community?		
**CHECK 111:**	**IF NOT SINCE BIRTH GO TO Q220**	**IF SINCE BIRTH SKIP TO Q221**	
220.	Thinking in terms of the community, where the people at the top (5) have the highest economic standing in that community and people at the bottom (1) have the lowest standing, where did you stand relative to other people in your **former** community?		
**Now I will like to ask you about something boys and girls do in this community**			
221.	Sometimes, some girls have sex in order to get money for their family or for themselves. Do you know of your neighbours' daughters who do this?<br>1=YES 2=NO 98=DON'T KNOW		
222.	Sometimes, some boys have sex in order to get money for their family or for themselves. Do you know of your neighbours' sons who do this?<br>1=YES 2=NO 8=DON'T KNOW		IF CODE 2 OR 8 SKIP TO Q224
223.	What is the main cause for your neighbours' daughters or sons having sex in exchange for money?<br>1=POVERTY 4=PEER PRESSURE/ BAD COMPANY<br>2= NO FOOD 5=LACK OF SELF DISCIPLINE<br>3=FAMILY/ MARITAL PROBLEMS 6=OTHER (SPECIFY)……………………………………………<br>8=DON'T KNOW		
224.	(a) Now let us talk about sex among teenagers in your community. Out of every ten girls in your community, how many would you say exchange sex for money?<br>(b) What about boys?<br>**IF DON'T KNOW,CODE 98**	**No. of girls**<br><br>**No. of boys**	
225.	Do you think that enough is done in this community to prevent young girls and boys from exchanging sex for money?<br>1=YES 2=NO 8=DON'T KNOW		
226.	What do you think is the best way to discourage young girls and boys in this community from exchanging sex for money?<br>1=FREE EDUCATION 2=INCOME GENERATING ACTIVITIES 3=SKILLS TRAINING<br>4=EDUCATION CAMPAIGNS 5=REDUCE FAMILY POVERTY<br>6=OTHER (SPECIFY)…………………………………………………………………………. 8=DON'T KNOW		
227.	Who should be mainly held responsible for the fact that some young girls/boys in your community get into this practice?<br>00=NOBODY 01=FATHER 02=MOTHER 03=BOTH MOTHER/FATHER<br>04=THE BOYS/GIRLS THEMSELVES 05=WHOLE COMMUNITY 06=THE GOVERNMENT<br>07=MEN/WOMEN WHO PAY THEM FOR SEX 08=OTHER (SPECIFY)………………………………………………<br>98=DON'T KNOW		

FLOODING			
228.	What are the effects of flooding on your community?<br>1=DESTROY HOUSES 2=POLLUTE DRINKING WATER 3=BRING ABOUT DISEASES 4=LOSS OF PROPERTY 5=LOSS OF HUMAN LIVES 6=OTHER (SPECIFY)..	☐	
229.	What do you think of the frequency of flooding now compared to the past 30 years?<br>1=INCREASED 2=DECREASED 3=SAME 8=DON'T KNOW	☐	
230.	Are you able to predict if there is going to be flooding?<br>1=YES 2=NO	☐	**If CODE 2 SKIP TO Q232**
231.	How are you able to predict that there is going to be flooding?<br>Explain..		

232.	What are the sources of support after flooding? (Probe: any other)<br>Code*<br>1=FINANCIAL 2= MATERIAL<br>3= SOCIAL 4= OTHER (SPECIFY)..........		**Yes**	**No**	**Support received***<br>**See code**	**IF "YES" TO GOVT OPTION, INSERT THE SUPPORT RECEIVED THEN SKIP TO Q234**
		a. Government				
		b. NGOs				
		c. Religious bodies				
		d. Family/Friends				
		e. Other(Specify)				

233.	What support do you want government to provide?<br>1=FINANCIAL 2=MATERIAL 3=SOCIAL 4=OTHER (SPECIFY)......................	☐	
234.	Do you receive information from Government institutions on flooding and storm?<br>1=YES 2=NO	☐	

235.	What can be done to prevent flooding in this community?<br>***(circle all the apply)***		**YES**	**NO**	
		a. AVOID BUILDING IN WATER WAY	1	2	
		b. CLEAN CLOGGED GUTTERS	1	2	
		c. CONSTRUCT WATER CHANNELS	1	2	
		d. OTHER (SPECIFY).............................	1	2	

236.	Will you consider relocating from this community because of the trend of flooding?<br>1=YES 2=NO 8=DON'T KNOW	☐	**If code 2 or 8, skip to next section**
237.	Where do you plan going to?	Name of community...	

SECTION 3: SHOULD BE ADMINISTERED TO MALE AND FEMALE RESPONDENTS.

SECTION 3: REPRODUCTIVE HEALTH

Now I would like to ask you some questions about your reproductive behaviour and reproductive health history. Remember that any information you provide will be kept strictly confidential. You may choose not to answer any of these questions.

301.	Do you listen to any radio shows or watch any TV show that discuss/ show sexual issues and situations?<br>1=YES 2. NO 8=DON'T KNOW/REMEMBER	☐	
302.	In the past 12 months have you watched a pornographic/blue film on TV, at a cinema hall, on a computer or elsewhere?<br>1=YES 2=NO	☐	IF CODE 2, SKIP TO Q304
303.	How did exposure to the film affect you?<br>……………………………………………………………………<br>……………………………………………………………………		
304.	When was the last time you kissed a member of the opposite sex?<br>**CODE 00 – IF NEVER. IF EVER, WRITE NUMBER OF DAYS/WEEKS/MONTHS AGO 98=DON'T KNOW**	**DAYS** ☐☐ **WEEKS** ☐☐ **MONTHS** ☐☐	
305.	When was the last time you fondled or were fondled by a member of the opposite sex? **CODE 00– IF NEVER. IF EVER, WRITE NUMBER DAYS/WEEKS/OF MONTHS AGO 98=DON'T KNOW**	**DAY** ☐☐ **WEEKS** ☐☐ **MONTHS** ☐☐	

306. a. Who would you talk to if you have problems about sex or sex concerns?

b. Who is the most likely person you would talk to? ***(circle the most likely)***

		Q306a		Q306b
		YES	NO	MOST LIKELY
a.	FATHER	1	2	a
i.	MOTHER	1	2	b
j.	HUSBAND/WIFE/PARTNER	1	2	c
k.	BROTHER	1	2	d
l.	SISTER	1	2	e
m.	UNCLE	1	2	f
n.	AUNT	1	2	g
o.	GRANDPARENT	1	2	h
p.	STEPMOTHER/FATHER	1	2	i
q.	OTHER RELATIVES	1	2	j
r.	FRIEND	1	2	k
s.	SCHOOL TEACHER	1	2	l
t.	RELIGIOUS LEADER	1	2	m
u.	COUNSELOR	1	2	n
v.	MEDICAL PERSON	1	2	o
w.	NO ONE	1	2	p
x.	OTHER (SPECIFY)	1	2	q

307.	Have you ever had sex?<br>1=YES 2= NO	☐	**IF CODE 2 SKIP TO Q336**

308. Who did you talk to about sex or sex concerns before your first sexual intercourse?

b. Who was the person you talked to most before your first sexual intercourse? ***(circle the most likely)***

		Q308a		Q308b
		YES	NO	MOST TALKED TO
a.	FATHER	1	2	a
b.	MOTHER	1	2	b
c.	HUSBAND/WIFE/PARTNER	1	2	c
d.	BROTHER	1	2	d
e.	SISTER	1	2	e
f.	UNCLE	1	2	f
g.	AUNT	1	2	g
h.	GRANDPARENT	1	2	h
i.	STEPMOTHER/FATHER	1	2	i
j.	OTHER RELATIVES	1	2	j
k.	FRIEND	1	2	k
l.	SCHOOL TEACHER	1	2	l
m.	RELIGIOUS LEADER	1	2	m
n.	COUNSELOR	1	2	n
o.	MEDICAL PERSON	1	2	o
p.	NO ONE	1	2	p
q.	OTHER (SPECIFY)	1	2	q

309. How old were you when you had your first sexual intercourse?

310. What was the **main circumstance** of your first sexual experience?

01. WAS PHYSICALLY FORCED INTO IT
02. IT WAS AN EXPRESSION OF MY/OUR LOVE
03. WAS PRESSURED INTO IT (FORCED BUT NOT PHYSICALLY)
04. WAS TRICKED INTO HAVING SEX
05. WANTED TO KNOW HOW IT FEELS
06. WANTED TO BE LIKE SOME OF MY FRIENDS
07. NEEDED MONEY
08. IN MARRIAGE
09. VOLUNTARY
96. OTHER (SPECIFY)……………………

311. In what setting did you have the first sexual intercourse?

01. PARENTS' HOUSE
02. PARTNER'S HOUSE
03. FRIEND'S HOUSE
04. HOTEL
05. IN A CAR
06. THE BUSH
07. SCHOOL CLASSROOM
08. SCHOOL DORMITORY
09. A TOILET/BATHROOM
10. A BACKYARD
11. THE STREET
12. AN ABANDONED BUILDING
13. OWN HOUSE
14. AT THE BEACH
96. OTHER (SPECIFY)……………………………………

312. How old was the person you had your first sexual intercourse with?

313. How was this person related to you?

01. HUSBAND/WIFE
02. FATHER/MOTHER
03. TOTAL STRANGER
04. STEPMOTHER/FATHER
05. STEPSIBLING
06. FAMILY FRIEND
07. BOYFRIEND/GIRLFRIEND
08. UNCLE/AUNT
09. COUSIN
10. OTHER RELATIVE
11. HOUSE GIRL /HOUSE BOY
12. NEIGHBOUR
13. CLASSMATE
14. TEACHER
15. RELIGIOUS LEADER
96. OTHER (SPECIFY)……………………………………

314. Were you given gifts or favours in return for your first sexual experience?

1=YES 2=NO

CHECK Q310 IF FORCED/PRESSURED/TRICKED CONTINUE ☐ **IF OTHERWISE SKIP TO Q321** ☐

I am sorry to bring up these memories which may or may not be painful or embarrassing to you, but could you please tell me if the person you had your first sexual encounter has done any of the following to you as well.

315. Did the person (adult or an older child) ask or demand you to......

	Q315a				Q315b
	**YES**	**NO**	**WON'T ANSWER**	**DON'T REMEMBER**	**MOST PAINFUL/ EMBARRASSING**
A) LOOK AT HIS/HER GENITALS?	1	2	3	8	A
B) UNDRESS AND SHOW HIM/HER YOUR GENITALS?	1	2	3	8	B
C) WATCH HIM/HER MASTURBATE?	1	2	3	8	C
D) UNDRESS WITH ANOTHER PERSON AND FONDLE EACH OTHER IN FRONT OF HIM/HER?	1	2	3	8	D
E) BE FONDLED (CARESSES, RUBS, KISSES ON THE WHOLE BODY AND/OR YOUR GENITALS)?	1	2	3	8	E
F) FONDLE HIM/HER (CARESSES, RUBS, KISSES ON THE WHOLE BODY AND/OR HIS/HER GENITALS)?	1	2	3	8	F
G) LOOK AT PORNOGRAPHIC PICTURES, DRAWINGS, FILMS, VIDEOTAPES OR MAGAZINES?	1	2	3	8	G
H) BE NAKED AND TO EXPOSE YOUR GENITALS FOR PICTURE TAKING OR FILMING?	1	2	3	8	H
I) ALLOW HIM/HER TO HAVE FULL SEXUAL INTERCOURSE WITH PENETRATION?	1	2	3	8	I
J) ALLOW HIM/HER TO HAVE HIS/HER FINGERS OR AN OBJECT INSERTED INTO YOUR PRIVATE PART?	1	2	3	8	J

316.	If you were subjected to one (or more) situation/s described in question above, how many times it happen to you with this person?			
317.	After this experience, did you feel……			

	YES	NO	DON'T REMEMBER
A. RESPONSIBLE FOR WHAT HAPPENED	1	2	8
B. THREATENED OR IN DANGER	1	2	8
C. EMBARRASSMENT OR SHAME	1	2	8
D. OTHER (SPECIFY) ………………………………………	1	2	8

318.	Did you discuss the experience with anyone?<br>1=YES 2=NO		IF CODE 2, SKIP TO Q321
319.	Who did you discuss the experience with?		

	YES	NO
A. FATHER	1	2
B. MOTHER	1	2
C. BROTHER/SISTER	1	2
D. GRANDPARENT	1	2
E. OTHER FAMILY MEMBER	1	2
F. SCHOOL NURSE	1	2
G. DOCTOR	1	2
H. FRIEND	1	2
I. OTHER (SPECIFY)	1	2

320. How did this person you told react to you? Were you ………..

	YES	NO
A. HELPED	1	2
B. NOT BELIEVED	1	2
C. ASKED TO KEEP IT A SECRET	1	2
D. TOLD NOT TO DO ANYTHING ABOUT IT	1	2
E. OTHER (SPECIFY)	1	2

ABORTION AND UNINTENDED PREGNANCIES

321.	Sometimes a girl becomes pregnant when she/her partner does not want her to. Have you ever been pregnant/made someone pregnant when you did not want to become pregnant/make someone pregnant?<br>1=YES 2=NO 3=NEVER BEEN PREGNANT/MADE SOMEONE PREGNANT		IF CODE 2, 3 SKIP TO Q324
322.	How many times has this occurred?	No.of Times	
323.	The last time this happened, did you want it then, later or not at all?<br>1=THEN 2=LATER 3=NOT AT ALL		
324.	Have you ever had an abortion/ made someone undergo an abortion?<br>1=YES 2=NO		IF CODE 2 SKIP TO Q326
325.	How many times have you had an abortion/made someone undergo abortion?	No. of Times	

**CHECK 100: IF FEMALE (CODE 1) GO TO Q326** ☐ **IF MALE (CODE 2) SKIP TO Q334** ☐			
326.	Now I would like to ask about all the births you had during your life. Have you ever had a live birth?<br>1=YES 2=NO	☐	**IF CODE 2 SKIP TO Q336**
327.	How many of these children are alive?<br>**IF NONE, RECORD '00'.**	☐☐	**IF CODE 00 SKIP TO Q332**
328.	a) How many sons live with you?<br>b) And how many daughters live with you?<br>**IF NONE, RECORD '00'.**	**SONS AT HOME** ☐☐<br>**DAUGHTERS AT HOME** ☐☐	
329.	Do you have any sons or daughters to whom you have given birth who are alive but do not live with you?<br>1=YES 2=NO	☐	**IF CODE 2 SKIP TO Q331**
330.	How many sons are alive but do not live with you? And how many daughters are alive but do not live with you?<br>**IF NONE, RECORD '00'.**	**SONS ELSEWHERE** ☐☐<br>**DAUGHTERS ELSEWHERE** ☐☐	
331.	Sometimes it happens that children die. This may be very painful to talk about and I am sorry to ask you about painful memories, but it is important to get the right information. Have you ever given birth to a child who was born alive but later died?<br>1=YES 2=NO<br>**NOTE: PROBE: ANY BABY WHO CRIED OR SHOWED SIGNS OF LIFE BUT SURVIVED ONLY A FEW HOURS OR DAYS?**	☐	**IF CODE 2, SKIP TO Q333**
332.	a) In all, how many boys have died?<br>b) And how many girls have died?<br>**IF NONE, RECORD '00'.**	**BOYS DEAD** ☐☐<br>**GIRLS DEAD** ☐☐	
333.	**SUM ANSWERS TO Q328, Q330 AND Q332 AND ENTER TOTAL.**<br>**IF NONE, RECORD '00'.**	☐☐	
**CHECK Q333:** Just to make sure that I have this right: you have had in TOTAL ______ children during your life. Is that correct?<br>1=YES 2=NO→**PROBE AND CORRECT Q328, Q330, Q332, Q333 AS NECESSARY**		☐☐	
334.	Now I would like to ask you about some current events in your life. Are you/your partner currently pregnant?<br>1=YES 2=NO 8=UNSURE	☐	**IF CODE 2, 8 SKIP TO Q336**
335.	For how many months have you/ your partner been pregnant?	**MONTHS** ☐☐	
**CHECK 326: HAS LIVING CHILDREN** ☐ **NO LIVING CHILDREN** ☐			
336.	**HAS LIVING CHILDREN**: If you could go back to the time you did not have any children and could choose exactly the number of children to have your whole life, how many would that be?<br>**NO LIVING CHILDREN**: If you could choose exactly the number of children to have in your whole life, how many would that be?<br>**PROBE FOR A NUMERIC RESPONSE**<br>OTHER (SPECIFY)…………………………….96	☐☐	

SECTION 4: SHOULD BE ADMINISTERED TO BOTH MALE AND FEMALE RESPONDENTS.

SECTION 4: CONTRACEPTION

Now I would like to talk about family planning – the various ways or methods that a couple can use to delay or avoid a pregnancy.

CIRCLE CODE 1 IN 401 FOR EACH METHOD MENTIONED SPONTANEOUSLY. IF NO MORE METHOD IS KNOWN, PROCEED TO Q402 AND ASK WHICH METHODS SHE HAS USED. PROMPT FOR MORE RESPONSES AND CIRCLE ALL MENTIONED.

Q. NO	QUESTIONS AND FILTERS	RESPONSE		SKIP
401.	Which ways or methods have you heard of?<br>**PROBE: ANY OTHER METHOD?**	**YES**	**NO**	
	a. PILL	1	2	
	b. IUD	1	2	
	c. INJECTIONS	1	2	
	d. IMPLANTS	1	2	
	e. DIAPHRAGM/FOAM/JELLY	1	2	
	f. MALE CONDOM	1	2	
	g. FEMALE CONDOM	1	2	
	h. FEMALE STERILIZATION	1	2	
	i. MALE STERILIZATION	1	2	
	j. NATURAL METHODS	1	2	
	k. WITHDRAWAL	1	2	
	l. LACTATIONAL AMENORRHEA (LAM)	1	2	
	m. OTHER (SPECIFY)………………………………………	1	2	
402.	Have you/your partner ever used anything or tried in any way to delay or avoid getting pregnant?<br>1=YES 2=NO		☐	**IF CODE 2, SKIP TO Q404**
403.	Which methods have you/your partner ever used?<br>**PROBE: ANY OTHERS?**	**YES**	**NO**	
	a. PILL	1	2	
	b. IUD	1	2	
	c. INJECTIONS	1	2	
	d. IMPLANTS	1	2	
	e. DIAPHRAGM/FOAM/JELLY	1	2	
	f. MALE CONDOM	1	2	
	g. FEMALE CONDOM	1	2	
	h. FEMALE STERILIZATION	1	2	
	i. MALE STERILIZATION	1	2	
	j. NATURAL METHODS	1	2	
	k. WITHDRAWAL	1	2	
	l. LACTATIONAL AMENORRHEA (LAM)	1	2	
	m. OTHER (SPECIFY)…………………………………………	1	2	

CHECK Q403: RESPONDENT NOT STERILIZED SKIP TO NEXT CHECK ☐
RESPONDENT STERILIZED SKIP TO Q408 ☐

CHECK 334: YOU/YOUR PARTNER NOT PREGNANT OR UNSURE GO TO Q404 ☐
YOU/ YOUR PARTNER PREGNANT SKIP TO Q408 ☐

Q. NO	QUESTIONS AND FILTERS	RESPONSE	SKIP
404.	Are you/your partner currently doing something or using any method to delay or avoid getting pregnant?<br>1=YES 2=NO	☐	**IF CODE 2 SKIP TO Q407**

405.	Which method are you/your partner using?	YES	NO	IF ONLY CODE 1 FOR I, j, k SKIP TO Q407
	a. PILL	1	2	
	b. IUD	1	2	
	c. INJECTIONS	1	2	
	d. IMPLANTS	1	2	
	e. DIAPHRAGM/FOAM/JELLY	1	2	
	f. MALE CONDOM	1	2	
	g. FEMALE CONDOM	1	2	
	h. FEMALE STERILIZATION	1	2	
	i. MALE STERILIZATION	1	2	
	j. NATURAL METHODS	1	2	
	k. WITHDRAWAL	1	2	
	l. OTHER (SPECIFY)……………………………………………	1	2	

406.	Where did you obtain (METHOD) the last time?<br>**(WRITE THE NAME OF THE PLACE AND AREA. PROBE TO IDENTIFY THE TYPE OF SOURCE AND ENTER THE APPROPRIATE CODE) – SPECIFY NAME OF PLACE AND AREA.**<br>**PUBLIC SECTOR………………………………………………………………………………………………**<br>11=GOVERNMENT HOSPITAL/POLYCLINIC 13=FAMILY PLANNING CLINIC<br>12=GOVERNMENT HEALTH CENTRE 14=MOBILE CLINIC<br>**MEDICAL PRIVATE SECTOR…………………………………………………………………………**<br>21=PRIVATE HOSPITAL OR CLINIC 22=PRIVATE DOCTOR<br>23=MOBILE CLINIC 24=FP/PPAG CLINIC<br>25=PHARMACY/DRUG STORE 31=FRIENDS/RELATIVES<br>96=OTHER (SPECIFY)--------------------------------------	☐☐	**SKIP TO Q408**

407.	What reasons do you have for not using an artificial method of contraception to avoid pregnancy?<br>**(PROBE: ANY OTHER REASONS?) RECORD ALL MENTIONED.**	YES	NO	MOST IMPORTANT	
	a. NOT MARRIED	1	2		
	b. INTEND TO MARRY	1	2		
	c. NOT INTEND TO MARRY	1	2		
	d. FERTILITY RELATED REASONS	1	2		
	e. OPPOSITION TO USE	1	2		
	f. LACK OF KNOWLEDGE	1	2		
	g. METHOD RELATED REASON	1	2		
	h. OTHER (SPECIFY)………………………………………………………	1	2		

408.	Have you obtained family planning services from any facility or healthcare person in the last 12 months?<br>1=YES 2=NO	☐	

SECTION 5: SHOULD BE ADMINISTERED TO ONLY FEMALE RESPONDENTS.

SECTION 5: CHILD HEALTH, NUTRITION AND FOOD SECURITY

ASK RESPONDENT : ONE OR MORE BIRTHS SINCE SEPTEMBER 2008 GO TO Q501 ☐
NO BIRTHS SINCE SEPTEMBER 2008 SKIP TO NEXT "ASK RESPONDENT" ☐

ENTER THE LINE NUMBER, NAME AND SURVIVAL STATUS OF THE LAST BIRTH BORN SINCE SEPTEMBER 2008 IN THE TABLE; ASK ALL THE QUESTIONS IN THIS SECTION IN REFERENCE TO THIS BIRTH.

Now I would like to ask you some more questions about the health of your last child born since September 2008

Q. NO	QUESTION	RESPONSE	SKIP
501.	LINE NUMBER FROM HH QUEST.	LAST BIRTH<br>Line number ☐☐	
502.	FROM HH QUEST.:	Name ……………………………………………<br>DEAD ☐ (IF DEAD SKIP TO Q515) ALIVE ☐	

ENTER LINE NUMBER, NAME AND SURVIVAL STATUS OF LAST BIRTH SINCE SEPTEMBER 2008 IN THE TABLE

Q.NO	QUESTION AND FILTER	RESPONSE	SKIP
503.	LINE NUMBER FROM HH QUEST.	**LAST BIRTH**<br>Line number ☐☐<br>NAME………………………………………	
504A.	Has (NAME) been ill with a fever at any time in the last 2 weeks?<br>1=YES 2=NO 8=DON'T KNOW	☐	**IF CODE 2, 8, SKIP TO Q505**
504B.	Did you seek medical advice or treatment for the fever?<br>1=YES 2=NO	☐	
505.	Has (NAME) been ill with a cough at any time in the last two weeks?<br>1=YES 2=NO 8=DON'T KNOW	☐	**IF CODE 2, 8, SKIP TO Q507**
506.	Did you seek medical advice or treatment for the cough?<br>1=YES 2=NO	☐	
507.	Has (NAME) had diarrhoea in the last two weeks?<br>1=YES 2=NO 8=DON'T KNOW	☐	**IF CODE 2,8, SKIP TO Q513**
508.	Was there any blood in the stools?<br>1=YES 2=NO 8=DON'T KNOW	☐	
509.	On the worst day of the diarrhoea, how many bowel movements did (NAME) have? **IF DON'T KNOW, ENTER 98**	**NO. OF BOWEL MOVEMENTS** ☐☐	
510.	Was anything given to treat the diarrhoea?<br>1=YES 2=NO 8=DON'T KNOW	☐	**IF CODE 2, 8, SKIP TO Q513**

Q.NO	QUESTION	YES	NO
511.	What was given to treat the diarrhoea?<br>**ANYTHING ELSE? CIRCLE ALL MENTIONED**		
	a. PILL OR SYRUP	1	2
	b. INJECTION	1	2
	c. INTRAVENOUS FLUID	1	2
	d. HOME REMEDIES/ HERBAL MEDICINES	1	2
	e. A FLUID MADE FROM A SPECIAL PACKET CALLED ORALITE OR ORS?	1	2
	f. THIN WATERY PORRIDGE MADE FROM MAIZE, RICE OR WHEAT?	1	2
	g. SOUP?	1	2
	h. HOMEMADE SUGAR-SALT-WATER SOLUTION?	1	2
	i. OTHER (SPECIFY)…………………………………………	1	2
512.	Did you seek medical advice or treatment for the diarrhoea?<br>1=YES 2=NO	☐	

ASK RESPONDENT: ARE YOU A CAREGIVER OF A CHILD(REN) UNDER 15 YEARS?
YES ☐ **→ Q513** **NO** ☐ **→ SKIP TO NEXT SECTION**

CLIMATE VARIABILITY AND HOUSEHOLD FOOD SECURITY (FOR CAREGIVERS OF CHILDREN UNDER 15 YEARS)			
513	Was your household present in the community during the 2011 October floods?<br>1= YES 2=NO	[ ]	IF CODE 1 SKIP TO Q515
514	After the 2011 October floods have you experience any other extreme climatic event in the community? 1=NO 2= YES, Floods 3=YES, Extreme high temperature<br>4= YES, Extreme low temperature 5= Other (Specify)_______________	[ ]	IF CODE 1 SKIP TO NEXT SECTION

I am going to read some statements that people have made about their food situation after the most recent extreme climatic event (e.g. floods in October 2011, droughts, high temperatures etc.). For these statements, please tell me whether they were: often true, sometimes true or never true for you or your household a month afterwards…

Response codes **01=often (always) true** **02=sometimes true** **03=never true**
98=don't know **00=refused to answer**

*****Questions 515 to 525 are in reference to the most recent extreme climatic event i.e. floods in 2011 / if not present in 2011 other extreme climatic event experience (specified in 514) after the 2011 October floods.**

515	"After extreme climatic event (eg. floods, drought, other event), we couldn't prepare the kind of foods we would want to eat for good health"<br>***Was that often true, sometimes true or never true for your household?***	[ ][ ]	
516	"After the extreme climatic event we were worried that our food would run out before we could get more"<br>***Was that often true, sometimes true or never true for your household?***	[ ][ ]	
517	"After the extreme climatic event the food that we bought just didn't last and we didn't have money to get more"<br>***Was that often true, sometimes true or never true for your household?***	[ ][ ]	
518	"After the extreme climatic event We couldn't afford to eat balanced meals."<br>***Was that often true, sometimes true, or never true for your household?***	[ ][ ]	
519	"After the extreme climatic event we were not able to feed our under 5 years old children the kinds of food we feel they needed to be healthy"<br>***Was that often true, sometimes true or never true for your household?***	[ ][ ]	
520	"After the extreme climatic event, Did **you and / or any adults in your household** ever reduce the amount of food they ate or skip meals because there wasn't enough food? 1=YES 2=NO	[ ]	**IF CODE 2 SKIP TO Q522**
521	How often did this happen a month after the extreme climatic event?<br>**1**=almost every day (4-7 days in a week)**2**=2-3 days in a week **3**=once a week or less<br>**8**=don't know	[ ]	
522	"After the extreme climatic event did your **children under 15 years old** ever eat less meals than usual because there wasn't enough food in the house?<br>1=YES 2=NO	[ ]	**IF CODE 2 SKIP TO Q524**
523	How often did this happen a month afterwards?<br>**1**=almost every day (4-7 days in a week)**2**=2-3 days in a week **3**=once a week or less<br>**8**=don't know	[ ]	
524	"After the extreme climatic event did any of the **children under 15 years old** *ever not eat for a whole day* because there wasn't enough food?<br>1=YES 2=NO	[ ]	**IF CODE 2 SKIP Q526**
525	How often did this happen a month after the extreme climatic event?<br>**1**=almost every day(4-7 days in a week) **2**=2-3 days in a week **3**=once a week or less<br>**8**=don't know	[ ]	

	CLIMATE VARIABILITY AND LIVELIHOODS		
**NO**	**QUESTION**	**RESPONSE**	

526. Which of the following extreme climatic events have your household experienced in the past 30 years? *(Tick all that apply)*		How would you rate the impact of this event on you or other household members on the following… ***(See Codes below for Q527 to Q530)**			
		Food Accessibility *(location you get food)*	Food Affordability (Price of food)	Food Availability (market availability)	Food Safety
		**Q527**	**Q528**	**Q529**	**Q530**
a. Floods					
b. Droughts					
c. Tropical storm					
d. High temperature					
e. Sea level rise					
f. Beach erosion					
g. Changes in rainy and dry seasons					
h. Higher sea water temp.					

Codes For Q527 to Q530*
1=Very severe 2=Severe 3=somewhat severe 4=little impact 5=No impact

531.	When did you experience the most recent extreme climatic event in this community?	(Month/Year) (_ _ _ _/ _ _ _ _ _)	
532.	What was the most recent extreme climatic event you experienced? 1=TROPICAL STORM 2=FLOODS 3=HIGH TEMP 4= SEA LEVEL RISE 5=OTHER (SPECIFY)……………….	☐	

IMPACT OF EXTREME CLIMATIC EVENT ON LIVELIHOODS

(Questions 533 to 568 are in relation to floods that occurred in 2011 or other extreme climatic event after the 2011 floods)

PHYSICAL

533.	Did the extreme climatic event cause any damage to any property/physical asset of yours? 1=YES 2=NO	☐	**IF CODE IS 2 SKIP TO Q535**
534.	Which physical assets of yours (household) was destroyed and to what extent?		

**Asset/property**	**1=YES 2=No/N.A**	**Slightly damaged**	**Moderately damaged**	**Severely damaged/ lost**
a. House/Shelter				
b. Furniture				
c. TV				
d. Working place /materials				
e. Fridge / freezer				
f. Other HHD assets_____				
g. Domestic animals				

535.	Did the extreme climatic event affect your household access to any community facility 1=YES 2=NO	☐	**IF CODE 2 SKIP TO Q537**

536.	Which facility did the extreme climatic event have an effect on, and to what extent did climatic event affect your accessibility?		
537.	Did the extreme climate event affect your household access to water? 1=YES 2=NO		IF CODE 2 SKIP TO Q539
538.	What was your source of water for the household during/after the extreme climatic event (Floods)<br>01=INDOOR PLUMBING<br>02=PRIVATE OUTSIDE STANDPIPE/TAP<br>03=RIVER/STREAM<br>04=INSIDE STANDPIPE<br>05=PUBLIC STANDPIPE<br>06= RAIN WATER/ SPRIN<br>07=WATER TRUCK/TANKER<br>08=BOLEHOLE<br>09=DUGOUT/POND/LAKE/DAM<br>10=WATER VENDOR<br>11=PROTECTED WELL<br>12=PIPE IN NEIGHBORING HOUSEHOLD<br>13=UNPROTECTED WELL<br>14=SACHET/BOTTLED WATER<br>15=OTHER SPECIFY_______		
539.	Did the extreme climate event affect your household toilet facility? 1=YES 2=NO		IF CODE 2 SKIP TO Q541
540.	How did the extreme climatic event affect your household toilet/sanitary facility?<br>………………………………………………………………………………………………<br>………………………………………………………………………………………………<br>………………………………………………………………………………………………		

Asset/property	1=YES 2=NO	Not at all accessible	Not accessible	accessible	Very accessible
A .Hospital /health center					
b. Schools					
c. Market					
d. Other________					

SOCIAL

541.	Did you or someone in your household relocate from the house or community because of the extreme climatic event (floods)? 1=YES 2=NO		
542.	Did your child(ren) under 15 years have to be taken care of by someone else in your community because of the extreme climatic event (floods)? 1=YES 2=NO		
543.	Did you have to **receive** any form of help from a friend /relatives/ group because of the extreme climatic event (floods)? 1=YES 2=NO		IF CODE IS 2 SKIP TO Q546
544.	From whom did you **receive** help?		

Individual / Organization	1=YES	2=NO
Relative		
Friend		
Government organization		
Non-governmental		
Other Specify		

545.	What type of help did you **receive** from individuals (Relatives and friends)		

Type of help	1=YES	2=NO
Money		
Food		
Medicine / health care		
Shelter		
Discussed about the event		
Other sp		

546.	Did you **give** any help to a friend / a group you belong to /relative after the extreme climatic event i.e. flood? 1=YES 2=NO		**IF CODE 2 SKIP TO Q548**
547.	What type of help did you **give** to individuals (Relatives and friends)?		

Type of help	1=YES	2=NO
a. Money		
b. Food		
c. Medicine / health care		
d. Shelter		
e. Discussed about the		
f. Other		

	**HUMAN**		**RESPONSE**
548.	Did you get injured or fall ill during or immediately after the extreme climatic event e.g. flood? 1=YES 2=NO		**IF CODE 2 SKIP TO Q550**
549.	What is / are the illness(s) you experience during or following the extreme climatic event (floods)? *(illness you attribute to the extreme climatic event*		

**Health condition**	**1=YES**	**2=NO**
a. Malaria		
b. DIARRHOEA		
c. CHOLERA		
d. SKIN RASH		
e. COUGH/DIPHTHERIA		
f. Other Specify________		

550.	Did any **ADULT** member of your household get injured or fall Sick during or immediately after the extreme climatic event e.g. flood? 1=YES 2=NO		**IF CODE 2 SKIP TO Q552**
551.	What was the main sickness you experience during or following the extreme climatic event (floods)?		

**Health condition**	1=YES	2=NO
a. Malaria		
b. Diarrhoea		
c. CHOLERA		
d. SKIN RASH		
e. COUGH/DIPHTHERIA		
f. Other Specify________		

552.	Did any **child under 15 years** in your household get injured or fall ill during or immediately after because of the extreme climatic event e.g.? flood? 1=YES 2=NO	

<table>
<tr><td>553.</td><td colspan="2">What was the main sickness or injury experience by the child(ren) under 15 years old during or following the extreme climatic event (floods)?

Health condition	1=YES	2=NO
Malaria		
DIARRHOEA		
CHOLERA		
SKIN RASH		
COUGH/DIPHTHERIA		
Other Specify________		

</td><td></td></tr>
<tr><td>554.</td><td colspan="2">Did the extreme climatic event (flood) affect your ability to work?
1=YES 2=NO</td><td>☐</td></tr>
<tr><td>555.</td><td>Did the recent extreme climatic event affect the way you care for your child(ren) under 15 years?
1=YES 2=NO</td><td>☐</td><td>IF CODE 2 SKIP TO Q557</td></tr>
<tr><td>556.</td><td colspan="3">Some caregivers have said the most climatic event affected the provision of care to their under 15 yr old children. It what extent do you agree this was applicable in your household

Activity/resource	Strong disagree	Disagree	Neutral	Agree	Strongly Agree
a. Spent less time with child					
b. Provided less food for your child					
c. Interrupted with the provision of school fees					
d. Provision of shelter					
e. Other(sp)________					

</td></tr>
<tr><td></td><td colspan="3">FINANCIAL</td></tr>
<tr><td>557.</td><td>Did the recent extreme climatic event affect your main income generated from you main occupation? 1=YES 2=NO</td><td>☐</td><td>IF CODE 1 SKIP TO Q559</td></tr>
<tr><td>558.</td><td colspan="2">How did the recent extreme climatic event affect your main IGA?
1= Reduced my income 4=Stopped my IGA
2=Increased my income 5=Bought it to a halt
3=Income remained the same 6=Other (sp)____________</td><td>☐</td></tr>
<tr><td>559.</td><td colspan="2">Did you have to use any savings or sell any property you had for the upkeep of the house? 1=YES 2=NO</td><td>☐</td></tr>
<tr><td>560.</td><td>Did the extreme climatic event make you go for a loan from a relative/ neighbour or organization? 1=YES 2=NO</td><td>☐</td><td>IF CODE 1 SKIP TO Q562</td></tr>
<tr><td>561.</td><td colspan="2">What was the main purpose of the money borrowed?
1=To buy food 4= For health care
2=To restore damage to property 5= Other (sp)______________________
3=For my Income generation activity</td><td>☐</td></tr>
<tr><td>562.</td><td colspan="2">If this climatic event e.g. flooding is to continue every year, what type of decision will you make concerning livelihood?
1= I will migrate 4=Do not know
2=Go for a wage labour work 5=Other (sp.)______________
3=Do other kinds of work</td><td>☐</td></tr>
</table>

563.	In your opinion, who in your household was the most affected by the extreme climatic even mentioned in Q526<br>1=MEN 2=WOMEN 3=CHILDREN	
564.	Give reason for who you believe is the most affected in your household?<br>--<br>--<br>____	

SECTION 6: SHOULD BE ADMINISTERED TO BOTH MALE AND FEMALE RESPONDENTS.

SECTION 6: MARRIAGE, SEXUAL ACTIVITY AND PARTNER'S BACKGROUND			
I would like to ask you some questions about marriage and will like to assure you that all information given me will be treated with strict confidentiality. You also have the right not to answer a question and it would not affect you in any way. Do I have your consent to continue the interview?			
**Q. NO**	**QUESTIONS AND FILTERS**	**RESPONSE**	**SKIP**
601.	OBSERVE PRESENCE OF OTHERS AT THIS POINT<br>**YES / NO**<br>a. CHILDREN UNDER 10 — 1 / 2<br>b. HUSBAND/PARTNER — 1 / 2<br>c. OTHER MALES — 1 / 2<br>d. OTHER FEMALES — 1 / 2		
602.	Are you currently married or living with a partner?<br>1=YES, CURRENTLY MARRIED 2=YES, LIVING WITH A PARTNER 3=NO, NOT IN UNION		**IF CODE 1, 2 SKIP TO Q607**
603.	Do you currently have a regular sexual partner, a casual sexual partner, an occasional sexual partner, or no sexual partner at all?<br>1=REGULAR SEXUAL PARTNER 2=OCCASIONAL SEXUAL PARTNER 3=NO SEXUAL PARTNER 4=CASUAL SEXUAL PARTNER		**IF CODE 3, 4 SKIP TO Q605**
604.	How long have you been in this relationship?<br>**IF LESS THAN A YEAR, RECORD IN MONTHS AND ENTER 00 FOR YEAR**	**MONTHS**<br>**YEARS**	
605.	Have you ever been married or lived with a man/woman?<br>1=YES EVER MARRIED 2=YES LIVED WITH A MAN/WOMAN 3=NO		**IF CODE 3, SKIP TO Q610**
606.	What is your marital status now?<br>1=WIDOWED 2=DIVORCED 3=SEPERATED		
607.	How long have you been married/living with your present spouse/partner?<br>**IF LESS THAN A YEAR, RECORD MONTH AND ENTER 00 FOR YEARS**	**MONTHS**<br>**YEARS**	
608.	Has any bridewealth been negotiated in this marriage?<br>1=YES<br>2=NO (PLEASE EXPLAIN)<br>……………………………………………………<br>……………………………………………………<br>……………………………………………………		**IF CODE 2, SKIP TO Q610**
609.	If yes, how much of it has been paid?<br>1= NOTHING PAID<br>2= PARTIALLY PAID<br>3= COMPLETELY PAID		
610.	What does bridewealth entitle one to?<br>……………………………………………………<br>……………………………………………………<br>……………………………………………………		
**CHECK Q605: IF CODE 3 GO TO SECTION 7**	**CHECK Q606:IF WIDOWED, DIVORCED OR SEPARATED SKIP TO Q614**		
611.	Is your spouse/partner living with you now or is he/she staying elsewhere?<br>1=LIVES WITH HIM/HER 2=STAYING ELSEWHERE		
612.	In your opinion, does your spouse/partner have any partner besides yourself?<br>1=YES 2=NO 8=DON'T KNOW		**IF CODE 2, 8 SKIP TO Q614**
613.	How many other spouses/partners does he/she have?<br>**IF DON'T KNOW, CODE 98**	**NUMBER**	

614.	Have you been married or lived with a man/woman only once, or more than once?<br>1=ONCE 2=MORE THAN ONCE		
**CHECK Q602:**	**CURRENTLY MARRIED OR LIVING WITH A PARTNER GO TO Q615**<br>**NOT IN UNION SKIP TO Q620**		
615.	**CHECK 401: MENTIONED CONDOM OR DID NOT MENTION CONDOM**<br>**MENTIONED CONDOM:** The last time you had sex with (your husband/the man you are living with), was a condom used?<br>1=YES 2=NO **IF DON'T REMEMBER, ENTER 8**<br>**DID NOT MENTION CONDOM:** Some men use a condom, which means that they use a rubber sheath on their penis during sexual intercourse. The last time you had sex with (your husband/ the man you are living with), was a condom used?<br>1=YES 2=NO **IF DON'T KNOW, ENTER 8**		
616.	Sometimes a man/woman may have sex with another person because circumstances force him/her to do so, or simply because they like the other person. Have you had sex with anyone other than your spouse/partner in the last 12 months?<br>1=YES 2=NO		**IF CODE 2, SKIP TO Q620**
617.	When was the last time you had sexual intercourse with someone other than (your spouse/partner you are living with)?	**DAYS AGO**<br>**WEEKS AGO**<br>**MONTHS AGO**	
618.	Was a condom used at that time?<br>1=YES 2=NO **IF DOES NOT KNOW, CODE 8**		
619.	How long has this been going on?<br>**IF RELATIONSHIP HAS ENDED, CODE 00**	**DAYS WEEKS MONTHS**	
620.	Now I need to ask you some questions about sexual activity in order to gain better understanding of some family planning issues. When was the last time you had sexual intercourse (if ever)?<br>**IF NEVER ENTER 00 (IN NEVER); IF LESS THAN ONE DAY, ENTER 00 (IN DAYS AGO); IF BEFORE LAST BIRTH, ENTER 96 (IN BEFORE LAST BIRTH)**	**NEVER**<br>**DAYS AGO**<br>**WEEKS AGO**<br>**MONTHS AGO**<br>**YEARS AGO**<br>**BEFORE LAST BIRTH**	
**CHECK Q620:**	**LESS THAN 12 MONTHS SINCE LAST SEX GO TO Q621**<br>**12 MONTHS OR LONGER SINCE LAST SEX SKIP TO NEXT CHECK**		
621.	In the last 12 months, how many persons have you had sex with?<br>**IF DON'T KNOW, CODE 98**	**NO. OF PERSONS**	
**CHECK Q602:**	**CURRENTLY MARRIED OR LIVING WITH A PARTNER GO TO Q622**<br>**NOT IN UNION SKIP TO NEXT SECTION**		
622.	Now, I am going to read you a series of statements. After I read each statement, please tell me whether you agree with the statement, disagree with it, or have no opinion one way or the other.<br>**1=AGREE 2=DISAGREE 3=NO OPINION**<br>a. If I ask my partner to use a condom, he/she would get angry or violent.<br>b. If I ask my partner to use a condom, he/she would think i'm having sex with other people.<br>c. My partner might be having sex with someone else.		

623.	Would your spouse/partner refuse your using modern contraceptives if you wanted to?<br><br>1=YES 2=NO 8=DON'T KNOW	☐	
624.	What would you do if he/she refused while you think you need the modern contraception?<br><br>1= I WOULD STILL USE IT<br>2=I WOULD NOT USE IT<br>3=I DON'T KNOW WHAT I WOULD DO<br>6=OTHER (SPECIFY)…………………………………………………………….	☐	

PARTNERS'S BACKGROUND

Now I would like to ask you questions about your partner's background and women's work and livelihood. Remember all information will be treated strictly confidential.

CHECK 602 AND 605: CURRENTLY MARRIED/LIVING WITH A PARTNER GO TO Q625 ☐
FORMERLY MARRIED/ LIVED WITH A PARTNER SKIP TO NEXT SECTION ☐
NEVER MARRIED AND NEVER IN A UNION SKIP TO NEXT SECTION ☐

625.	How old was your spouse/partner on his last birthday?<br><br>**IF DON'T KNOW CODE 98**	☐☐	
626.	Is your spouse/partner much younger, a little younger, about the same age, a little older or much older than you?<br><br>1=MUCH YOUNGER 2=A LITTLE YOUNGER 3=ABOUT THE SAME AGE<br>4=A LITTLE OLDER 5=MUCH OLDER	☐	
627.	What was the highest level of education he/she attended?<br><br>0=NO EDUCATION 1=PRE-SCHOOL 2=PRIMARY 3=JSS/MIDDLE<br>4=SSS/SECONDARY 5=HIGHER 8=DON'T KNOW	☐	**IF CODE 0,1, 8, SKIP TO Q629**
628.	What is the highest grade he/she completed at that level?<br><br>**IF DON'T KNOW, CODE 98**	☐☐	
629.	What is (was) your spouse/partner's occupation? That is, what kind of work does (did) he/she ***mainly*** do?<br><br>NAME OF OCCUPATION……………………………………………………………………….<br><br>01=NO OCCUPATION 07=HOUSEHOLD AND DOMESTIC<br>02=PROFESSIONAL/TECHNICAL/MANAGEMENT 08=SERVICE<br>03=CLERICAL 09=SKILLED MANUAL<br>04=SALES 10=UNSKILLED MANUAL<br>05=AGRICULTURE- SELF EMPLOYED 11=OTHER (SPECIFY)……………………………………….<br>06=AGRICULTURE 98=DON'T KNOW	☐☐	

SECTION 7: SHOULD BE ADMINISTERED TO BOTH MALE AND FEMALE RESPONDENTS.

<table>
<tr><th colspan="4">SECTION 7: GENERAL PHYSICAL HEALTH AND RELATED ISSUES</th></tr>
<tr><th colspan="4">DIARRHOEA</th></tr>
<tr><td>701.</td><td>How many times did you have diarrhoea within the past five years?</td><td>☐☐☐</td><td></td></tr>
<tr><td>702.</td><td>In your opinion, what do you think about the incidence of diarrhoea over the past five years?
1. INCREASING
2. DECREASING
3. REMAINS THE SAME
4.DON'T KNOW</td><td>☐</td><td></td></tr>
<tr><td>703.</td><td>In your opinion, does diarrhoea incidence in your community increase with the number of rainy days?
1=YES 2=NO 8=DON'T KNOW</td><td>☐</td><td>IF CODE 2, 8 SKIP TO Q706</td></tr>
<tr><td>704.</td><td>Do you take any measure to prevent diarrhoea anytime it rains?
1=YES 2=NO</td><td>☐</td><td>IF CODE 2 SKIP TO Q706</td></tr>
<tr><td>705.</td><td colspan="2">Which specific measures do you take?
……………………………………………………………………………………………………
……………………………………………………………………………………………………</td><td></td></tr>
<tr><th colspan="4">MALARIA</th></tr>
<tr><td colspan="4">Briefly explain Malaria to Respondent

Malaria is caused by a bite from an infected female Anopheles mosquito which introduces the protists (a type of microorganism) through saliva into the circulatory system. In the blood, the protists travel to the liver to mature and reproduce. Malaria causes symptoms that typically include fever, feeling cold, and headache, bitter taste in the mouth, etc.</td></tr>
<tr><td>706.</td><td>How many times did you have malaria within the past five years?</td><td>☐☐</td><td></td></tr>
<tr><td>707.</td><td>In your opinion, what do you think about the incidence of malaria over the past five years?
1. INCREASING
2. DECLINING
3. REMAINS THE SAME</td><td>☐</td><td></td></tr>
<tr><td>708.</td><td>Which of the following rainy situations has your community experienced over the past five years?
1. INCREASING NUMBER OF RAINY DAYS EACH YEAR
2. DECREASING NUMBER OF RAINY DAYS EACH YEAR
3. SAME NUMBER OF RAINY DAYS PER YEAR</td><td>☐</td><td></td></tr>
<tr><td>709.</td><td>In your opinion, does malaria incidence in your community increase with the number of rainy days?
1=YES 2=NO 8=DON'T KNOW</td><td>☐</td><td>IF CODE 2 AND 8, SKIP TO Q712</td></tr>
<tr><td>710.</td><td>Do you take any measure to prevent malaria anytime it rains?
1=YES 2=NO</td><td>☐</td><td>IF CODE 2 SKIP TO Q712</td></tr>
<tr><td>711.</td><td colspan="2">Which specific measures do you take?
……………………………………………………………………………………………………..
……………………………………………………………………………………………………</td><td></td></tr>
<tr><td>712.</td><td colspan="2">Indicate whether or not the following can cause malaria.
<table><tr><th></th><th>YES</th><th>NO</th></tr><tr><td>a. DRINKING DIRTY WATER</td><td>1</td><td>2</td></tr><tr><td>b. WITCHCRAFT</td><td>1</td><td>2</td></tr><tr><td>c. STANDING WATER</td><td>1</td><td>2</td></tr></table></td><td></td></tr>
<tr><td>713.</td><td>When was the last time you or any member of your household had malaria?
1=LESS THAN A WEEK AGO 2=A WEEK AGO 3=TWO WEEKS AGO
4=THREE WEEKS AGO 5=A MONTH AGO 6=MORE THAN A MONTH AGO
7= NEVER</td><td>☐</td><td></td></tr>
</table>

714.	How many times did you or any member of your household suffer from malaria in the past one year?<br>**Code 00 if Never**	☐☐		
715.	If **you or any member of your family** ever had malaria, which of the following symptoms did you experience?<br>***(circle all that apply)***	**YES**	**NO**	
	a. FEVER	1	2	
	b. FEELING COLD/CHILLS/SHIVERING	1	2	
	c. BODY ACHES/JOINT PAINS/WEAKNESS	1	2	
	d. BITTER TASTE	1	2	
	e. LOSS OF APPETITE	1	2	
	f. VOMITING	1	2	
	g. HEADACHE	1	2	
	h. NAUSEA	1	2	
	i. VAGUE FEELING/RESTLESSNESS	1	2	
	j. SLEEPLESSNESS	1	2	
	k. PALE LOOKING	1	2	
	l. PERSPIRATION	1	2	
	m. YELLOWISH PALM	1	2	
	n. DELIRIUM (BAD BREATH)	1	2	
	o. YELLOW EYE BALL	1	2	
	P.OTHER (SPECIFY)___________________________	1	2	
716.	How many days on the average do you experience the symptoms of malaria before you begin any treatment?	**DAYS** ☐☐		
717.	When you have malaria, which of the following treatments do you seek first?<br>1. HERBAL TREATMENT<br>2. TRADITIONAL MEDICINE MAN<br>3. DRUG STORE/PHARMACY<br>4. PRIVATE CLINIC/HOSPITAL<br>5. PUBLIC/GOVERNMENT HOSPITAL OR HEALTH POST<br>6. FAITH HEALER<br>7. SELF PRESCRIPTION<br>8. Other specify_____________________________	☐		
718.	If you ever visited the pharmacy for malaria drugs, how much on the average do you spend on it?	AMOUNT IN GH₵		PESEWAS
719.	If your first treatment option is not a visit to a hospital or health post, how long after you start self-medication do you decide to visit the hospital?	**DAYS** ☐☐		
720.	Which of the following is the most common breeding site of mosquitoes in your area?<br>1. RUNNING DIRTY WATER<br>2. GARBAGE/TRASH<br>3. STANDING CLEAN WATER<br>4. STANDING DIRTY WATER<br>5. RUNNING CLEAN WATER<br>6 PLANTS/VEGETATION AROUND THE HOUSE<br>8. NO IDEA/DON'T KNOW	☐		
721.	When you are sick of malaria, how many days on the average does it take you to fully recover?	**DAYS** ☐☐		
722.	Do you know any individual who died from malaria within the last year?<br>1 = YES 2 = NO	☐		

723.	Within the last month, how many days did you take the following preventive measures against malaria?<br><br>a. USE OF SMOKE TO DRIVE AWAY MOSQUITOES<br>b. MOSQUITO COILS<br>c. MOSQUITO SPRAY<br>d. MOSQUITO REPELLING CREAM<br>e. USE OF FAN<br>f. COVERING OF BODY WITH CLOTHES<br>g. MOSQUITO NET<br>h. MOSQUITO PROOF WINDOWS<br>i. OTHERS (SPECIFY)…………………	**Number of days within last month**	
724.	If someone uses the following preventive measures each day of the month, how will you rate his/her chances of suffering from malaria? Please use the following scale:<br>1= VERY HIGH (AT LEAST 1 IN 2), 2=HIGH (AROUND 1 IN 4), 3= QUITE POSSIBLE (1 IN 10), 4= MODERATELY LOW (1 IN 20),<br>5=VERY LOW (AT MOST 1 IN 100)<br>**Practice**<br>a. USE OF SMOKE TO DRIVE AWAY MOSQUITOES<br>b. MOSQUITO COILS<br>c. MOSQUITO SPRAY<br>d. MOSQUITO REPELLING CREAM<br>e. USE OF FAN<br>f. COVERING OF BODY WITH CLOTHES<br>g. MOSQUITO NET<br>h. MOSQUITO PROOF WINDOWS	**Indicate chance (use scale)**	
725.	What is your health insurance enrolment status?<br>1. Currently enrolled in the NHIS<br>2. Previously enrolled in the NHIS<br>3. Never enrolled in the NHIS<br>4. Different health insurance scheme (specify)…….		
726.	What is the distance from your residence to your regular hospital?<br>**IF NEVER, CODE 999**	**DISTANCE**<br>**KM** **MINUTES**	**IF CODE 999, SKIP TO Q731**
727.	On the average, how much is the return transportation cost to the hospital?	**AMOUNT GH₵**	**PESEWAS**
728.	How long do you usually wait at the hospital before you are able to see a doctor?	**MINUTES**	
729.	How much on the average do you spend at the hospital when you seek malaria treatment?	**AMOUNT GH₵**	**PESEWAS**
730.	Have you been hospitalized within the last one year as a result of malaria?<br>1 = YES 2 = NO		

731.	**Discount Rates Using Matching and Choice Experiment**<br><br>This part of the questionnaire involves making some choices between two alternatives. Please think carefully before you answer.<br><br>**(A)** Suppose your District Assembly wants to implement project **A** or **B** in your District. The two projects cost the same amount of money. Which of the following project will you vote for?<br>Project **A** would increase your income once by GHS100 by the end of this month (i,e., September)<br><br>Project **B** would increase your income once by GHS 200 at the end of 6 months (i.e., February)<br><br>**(B)** If you are to quote a value for alternative **B** that will make you exactly as happy as choosing alternative **A,** what value will that be? And vice versa.	**AMOUNT GH₵ PESEWAS**	
732.	**The Risk Experiment**<br>We would now like to know how you would choose between savings from different malaria prevention programs. There are equal chances (50%) of good or bad outcomes from the program. (Exemplify with a coin that is tossed: head representing bad outcome and tail representing good outcome).<br>1 = TOOK THE RISK (chose the coin toss) 2=DID NOT TAKE THE RISK (chose the expected mean)		

	Bad outcome GHÇ	Good outcome GHÇ	Expected mean GHÇ	Risk Taking
Choice set 1	10	10	10	
Choice set 2	9	18	13.50	
Choice set 3	8	24	16	
Choice set 4	6	30	18	
Choice set 5	2	36	19	
Choice set 6	0	40	20	

The Contingent Valuation Experiment

Suppose the ministry of health is considering reducing malaria incidence in your community. The ministry will use a chemical called Fenthion (82.5% w/v). Fenthion is a compound with quick killing action on larvae with long residual effect. The chemical is mainly applicable to polluted water in ditches, ponds, swamps, septic tanks and other mosquito breeding sites that are not used as drinking water by humans or domestic animals. The frequency of application is once a week and it is expected that this will reduce malaria incidence by 20%. This may have to be decreased or increased on the preliminary observations in index breeding places depending upon the residual effect of the larvicide. Note that the chemical will kill the larvae at location where it has been applied but will not kill mosquitoes that already exist in the community or may travel from other communities.

The cost involved in spraying the chemicals include the cost of the chemical, hand compression sprayer, water to be mixed with the chemical, and labour time. Government does not have the budget to support this initiative. Your community may have to support it by contributing either labour, time, money or both time and money.

733.	Will you be willing to contribute money or your time or both to support such an initiative? 1= YES 2 = NO		**IF YES SKIP TO Q735**
734.	If NO,<br>please explain why..<br>..		**SKIP Q738**
**CHECK 733: IF CODE 1 ANSWER Q735**		**IF CODE 2 SKIP TO Q738**	
735.	If you are willing to contribute time, what is the maximum amount of time you are willing to contribute each month?	AMOUNT OF TIME (in hours)	**IF 'TIME ONLY' SKIP TO Q738**

736.	If you are willing to contribute money, what is the maximum amount that you are willing to contribute each month? The donation is supposed to be given directly to an official from the ministry of health who will visit your house each month to collect it.	AMOUNT IN GH₵	
737.	This question is about trust: If you pay money for such an initiative, how do you rate the chances that the money will be used for the intended purpose?<br>1. VERY HIGH (AT LEAST 1 IN 2),<br>2. HIGH (AROUND 1 IN 4),<br>3. QUITE POSSIBLE (1 IN 10),<br>4. MODERATELY LOW (1 IN 20),<br>5. VERY LOW (AT MOST 1 IN 100)		

738. **THE CHOICE EXPERIMENT**

An anti-malaria drug (e.g., artesunate-amodiaquine) is either manufactured in Ghana, imported from India or Belgium, among other countries. A test on samples of the drug reveals varying degrees of active ingredients and, consequently, cures rates depending on which of the three countries produced it. Suppose some of the samples had cure rates which were 5% and 10% lower than the expected rate. In addition, the retail price of the drug ranges from GHS 2.00 to GHS 5.00. A decision to purchase the drug has to take into consideration the place of manufacture, the cure rate, and the price.

Do you have any questions regarding the preceding narrative? **(PROMPT)**

Before we go to questions regarding your actual choice, please review the attributes.

Attribute	Level
Change in cure rate	*-5%*<br>*-10%*
Country of Origin	*Belgium*<br>*India*<br>*Ghana*
Prices	*GHS 2.00*<br>*GHS 3.00*<br>*GHS 5.00*

For each of the following tables, please indicate your choice.

Choice Set 1

	Option 1	Option 2	Option 3 (Opt out)
Origin	Indian Brand	Ghana Brand	
Change in cure rate	-10%	-5%	
Price	GH₵3.00	GH₵3.00	

Choice Set 2

	Option 1	Option 2	Option 3 (Opt out)
Origin	Ghana Brand	Ghana Brand	
Change in cure rate	-5%	-10%	
Price	GH₵3.00	GH₵2.00	

Choice Set 3

	Option 1	Option 2	Option 3 (Opt out)
Origin	Belgium Brand	Ghana Brand	
Change in cure rate	-10%	-5%	
Price	GH₵3.00	GH₵3.00	

Choice Set 4

	Option 1	Option 2	Option 3 (Opt out)
Origin	Indian Brand	Belgium Brand	
Change in cure rate	-10%	0%	
Price	GH₵3.00	GH₵5.00	

739.	On which occasion(s) did you wash your hands yesterday? ***Probe only once***	**MENTIONED**	**NOT MENTIONED**	**WITH SOAP**	
	A = DID NOT WASH HANDS YESTERDAY	1	2	A	
	B = BEFORE PREPARING FOOD	1	2	B	
	C = BEFORE EATING FOOD	1	2	C	
	D = BEFORE FEEDING CHILD	1	2	D	
	E = BEFORE PRAYER	1	2	E	
	F = AFTER CLEANING THE CHILD'S BOTTOMS	1	2	F	
	G = AFTER COMING FROM WASHROOM	1	2	G	
	H = AFTER EATING	1	2	H	
	I = AFTER FEEDING CHILD	1	2	I	
	J = AFTER PREPARING FOOD	1	2	J	
	K = AFTER CLEANING HOUSE	1	2	K	
	L = WASH HANDS AND FACE	1	2	L	
	M = HAVING/GIVING BATH	1	2	M	
	N = CLEAN UTENSILS	1	2	N	
	O = WASHING CLOTHES	1	2	O	
	P = OTHER (SPECIFY)____________________	1	2	P	

SECTION 8: SHOULD BE ADMINISTERED TO BOTH MALE AND FEMALE RESPONDENTS.

SECTION 8: NON-COMMUNICABLE DISEASES AND PHYSICAL ACTIVITY

SECTION 8A: CHRONIC NON-COMMUNICABLE DISEASE CONDITIONS

Now we are going to talk about chronic non-communicable disease conditions

801.	Have you ever heard of any of the following:	YES	NO	Sources of information* (USE CODES IN Q802)	
	a. HEART DISEASE (ANGINA, ABNORMAL HEART RHYTHM)?	1	2		IF NO TO ALL SKIP TO Q810
	b. STROKE?	1	2		
	c. DIABETES?	1	2		
	d. CHRONIC LUNG DISEASE (CHRONIC BRONCHITIS OR EMPHYSEMA)?	1	2		
	e. HYPERTENSION (HIGH BLOOD PRESSURE)?	1	2		
	f. CANCER OR A MALIGNANT TUMOR (BREAST, PROSTATE, ETC.)?	1	2		
	g. ASTHMA?	1	2		
	h. ARTHRITIS?	1	2		
	i. KIDNEY DISEASE?	1	2		
	j. LIVER DISEASE?	1	2		
	k. HIGH BLOOD CHOLESTEROL	1	2		
	l. OTHER (SPECIFY)	1	2		

802. From which sources of information did you hear about the diseases above? Any other source?
PROBE: **ANY OTHER? RECORD ALL RESPONSES IN SPACE PROVIDED IN Q801**

1= TV
2=RADIO
3= NEWSPAPERS/MAGAZINES
4= PAMPLETS/POSTERS
5= HEALTH WORKERS
6= MOSQUES/CHURCHES
7= SCHOOLS/TEACHERS
8= COMMUNITY MEETINGS
9= FRIENDS /RELATIVES
10= WORK PLACE
11= DRAMA/PERFORMANCE
96= OTHER (specify)...................................

803. a. Have you ever been told/diagnosed by a medical professional that you have any of these conditions? ***(circle all that apply)***
b. How long ago were you diagnosed?

	Q803a		Q803b		IF NO IN ALL SKIP TO Q809
	YES	NO	If yes, how long ago (years)?		
a. HEART DISEASE (ANGINA, ABNORMAL HEART RHYTHM)?	1	2			
b. STROKE?	1	2			
c. DIABETES?	1	2			
d. CHRONIC LUNG DISEASE (CHRONIC BRONCHITIS OR EMPHYSEMA)?	1	2			
e. HYPERTENSION (HIGH BLOOD PRESSURE)?	1	2			
f. CANCER OR A MALIGNANT TUMOR (eg. BREAST, PROSTATE)?	1	2			
g. ASTHMA?	1	2			
h. ARTHRITIS?	1	2			
i. KIDNEY DISEASE?	1	2			
j. LIVER DISEASE?	1	2			
k. HIGH BLOOD CHOLESTEROL	1	2			
l. OTHER (SPECIFY)	1	2			

804.	The first time you were told/diagnosed (disease(s) from **Q803**) did you seek advice or treatment?<br>1=YES 2=NO	☐	**IF CODE 2 SKIP TO Q810**

805.	Where did you seek advice or treatment? Any other place or person?<br>**MENTION RESPONSES TO RESPONDENT AND RECORD ALL RESPONSES**			
		**YES**	**NO**	
	a. GOVERNMENT HOSPITAL	1	2	
	b. GOVERNMENT HEALTH CENTRE	1	2	
	c. GOVERNMENT DISPENSARY	1	2	
	d. MISSION HOSPITAL/CLINIC	1	2	
	e. OTHER PRIVATE HOSPITAL/CLINIC	1	2	
	f. PHARMACY	1	2	
	g. PRIVATE DOCTOR	1	2	
	h. MOBILE CLINIC	1	2	
	i. COMMUNITY BASED DISTRIBUTOR	1	2	
	j. COMMUNITY HEALTH WORKER	1	2	
	k. SHOP	1	2	
	l. HERBALIST/TRADITIONAL PRACTITIONER	1	2	
	m. HERBAL CLINIC	1	2	
	n. FETISH PRIEST	1	2	
	o. CHINESE MEDICINE	1	2	
	p. CHURCH /FAITH HEALING	1	2	
	q. RELATIVE/FRIEND	1	2	
	r. OTHER (SPECIFY).................................	1	2	
	s. DOES NOT KNOW	1	2	
806.	Are you taking any medication or therapy for the condition (Check for condition in Q803)... during the last **two weeks**?			
		**YES**	**NO**	
	a. HEART DISEASE (ANGINA, ABNORMAL HEART RHYTHM)?	1	2	
	b. STROKE?	1	2	
	c. DIABETES?	1	2	
	d. CHRONIC LUNG DISEASE (CHRONIC BRONCHITIS OR EMPHYSEMA)?	1	2	
	e. HYPERTENSION (HIGH BLOOD PRESSURE)?	1	2	
	f. CANCER OR A MALIGNANT TUMOR (BREAST, PROSTATE, ETC.)?	1	2	
	g. ASTHMA?	1	2	
	h. ARTHRITIS?	1	2	
	i. KIDNEY DISEASE?	1	2	
	j. LIVER DISEASE?	1	2	
	k. HIGH BLOOD CHOLESTEROL	1	2	
	l. OTHER (SPECIFY)……………………………………….	1	2	
807.	Are you taking any medication or therapy for the condition (Check for condition in Q803)... during the last **12 month**?			
		**YES**	**NO**	
	a. HEART DISEASE (ANGINA, ABNORMAL HEART RHYTHM)?	1	2	
	b. STROKE?	1	2	
	c. DIABETES?	1	2	
	d. CHRONIC LUNG DISEASE (CHRONIC BRONCHITIS OR EMPHYSEMA)?	1	2	
	e. HYPERTENSION (HIGH BLOOD PRESSURE)?	1	2	
	f. CANCER OR A MALIGNANT TUMOR (BREAST, PROSTATE, ETC.)?	1	2	
	g. ASTHMA?	1	2	
	h. ARTHRITIS?	1	2	
	i. KIDNEY DISEASE?	1	2	
	j. LIVER DISEASE?	1	2	
	k. HIGH BLOOD CHOLESTEROL	1	2	
	l. OTHER (SPECIFY)………………………………………………….	1	2	

808.	During the past 4 weeks, how much did illness interfere with your normal work (including both work outside the home and housework)?<br><br>0=NOT AT ALL 1= A LITTLE BIT 2=MODERATELY 3= QUITE A BIT 4= EXTREMELY		
**CHECK 803: IF NO TO ALL GO TO Q809**		**IF AT LEAST ONE YES SKIP TO Q810**	
809.	Do you think your chances of getting any of the following diseases is<br>**1=SMALL 2=MODERATE 3=GREAT 4=NO RISK AT ALL**		
	a. HEART DISEASE (ANGINA, ABNORMAL HEART RHYTHM)?	a	
	b. STROKE?	b	
	c. DIABETES?	c	
	d. CHRONIC LUNG DISEASE (CHRONIC BRONCHITIS OR EMPHYSEMA)?	d	
	e. HYPERTENSION (HIGH BLOOD PRESSURE)?	e	
	f. CANCER OR A MALIGNANT TUMOR (BREAST, PROSTATE, ETC.)?	f	
	g. ASTHMA?	g	
	h. ARTHRITIS?	h	
	i. KIDNEY DISEASE?	i	
	j. LIVER DISEASE?	j	

CVD KNOWLEDGE SCALE

810. The questions below ask about your health related issues. Please select an answer for each question. If you agree with the statement choose 2; if you don't agree, choose 1; and if you are not sure, choose 0

AGREE = 2 DISAGREE = 1 NOT SURE = 0

a.	Heart disease can be prevented		
b.	Cigarette smoking can cause heart disease and stroke.		
c.	There is greater risk for heart disease in elderly		
d.	People with high blood pressure should avoid salt in their diets.		
e.	If people quit smoking it reduces the risk of heart disease		
f.	Salty food makes causes high blood pressure		
g.	High blood pressure is a risk factor for heart disease		
h.	Fatty foods do not increase blood cholesterol levels		
i.	More than 3 meals per week of red meat is not good for your health		
j.	Eating fruit and vegetables every day is beneficial		
k.	Overweight people are more likely to have heart disease		
l.	Regular exercise reduces risk of heart disease		
m.	Regular walking can reduce risk of heart disease		
n.	Stress and sadness increase the risk of heart disease		
o.	Under stressful situations, blood pressure will increase		
p.	To keep blood pressure under control will reduce the risk of heart disease		
q.	People with high blood pressure need to use blood pressure medicine for life		
r.	Heavy alcohol use does not affect your blood pressure.		
s.	High cholesterol is a risk factor for heart disease		
t.	People with high cholesterol need to take medication		
u.	Diabetes is a risk factor for heart disease		
v.	People with diabetes need to control sugar intake		
w.	Heart disease in your family will increase your risk of heart disease		
x.	Shortness of breath may be a sign of heart disease.		
y.	Feeling chest pain or discomfort can be a sign of heart disease		
z.	People should only get their blood pressure checked if they have chest pain or headaches		
aa.	If you have high blood pressure taking a medicine for one month can cure you		

SECTION 8B: PHYSICAL ACTIVITY AND BODY SHAPE			
CHECK Q142, IF NOT WORKING SKIP TO Q817			
Please describe your physical activity at work			
811.	Does your work involve vigorous-intensity activity that causes large increases in breathing or heart rate like (carrying or lifting heavy loads, digging or construction work) for at least 10 minutes continuously<br>1=YES 2=NO		IF CODE 2 SKIP TO Q814
812.	In a typical week, on how many days do you do vigorous-intensity activities as part of your work?	Days	
813.	How much time do you spend doing vigorous-intensity activities at work on a typical day?	Hours<br>Minutes	
814.	Does your work involve moderate-intensity activity that causes small increases in breathing or heart rate such as brisk walking (or carrying light loads) for at least 10 minutes continuously?<br>1=YES 2=NO		IF CODE 2 SKIP TO Q817
815.	In a typical week, on how many days do you do moderate-intensity activities as part of your work?	Number of days	
816.	How much time do you spend doing moderate-intensity activities at work on a typical day?	Hours<br>Minutes	
Please describe your physical activity when you travel to and from places			
817.	Do you walk or use a bicycle (pedal cycle) for at least 10 minutes continuously to get to and from places?<br>1=YES 2=NO		IF CODE 2 SKIP TO Q820
818.	In a typical week, on how many days do you walk or cycle for at least 10 minutes to get to and from places?	Days	
819.	How much time do you spend walking or cycling for travel on a typical day?	Hours<br>Minutes	
Please describe your physical activity during your recreational activities			
820.	Do you do any vigorous-intensity sports, fitness or recreational (leisure) activities that cause large increases in breathing or heart rate (like running or football) for at least 10 minutes continuously? 1=YES 2=NO		IF CODE 2 SKIP TO Q823
821.	In a typical week, on how many days do you do vigorous-intensity sports, fitness or recreational (leisure) activities?	Days	
822.	How much time do you spend doing vigorous-intensity sports, fitness or recreational activities on a typical day?	Hours<br>Minutes	
823.	Do you do any moderate-intensity sports, fitness or recreational (leisure) activities that cause a small increase in breathing or heart rate such as brisk walking, (cycling, swimming, volleyball) for at least 10 minutes continuously?<br>1=YES 2=NO		IF CODE 2 SKIP TO Q826
824.	In a typical week, on how many days do you do moderate-intensity sports, fitness or recreational (leisure) activities?	Days	
825.	How much time do you spend doing moderate-intensity sports, fitness or recreational (leisure) activities on a typical day?	Hours<br>Minutes	

Please describe your physical activity on a usual day (sedentary behaviour)			
826.	How much time do you usually spend sitting or reclining on a typical day? *This question refers to the day time.*	Hours ☐☐<br>Minutes ☐☐	
827.	During an average week, how much time do you spend sitting or lying down while watching TV, videos, or DVDs?<br>1=0-1 HOURS<br>2=2-5 HOURS<br>3=6-20 HOURS<br>4=21-40 HOURS<br>5=OVER 40 HOURS	☐	

SECTION 8C: BODY SHAPE

BODY SHAPE – QUESTIONS FOR WOMEN ONLY

828. We want to ask some questions about body shape. For the following questions, you can choose one of the pictures below. Under each picture is a number. Please use this number for your answer.

Place an **X** under one of the numbers below:

	1	2	3	4	5	6	7	8	9
a. Which picture do you most look like right now?									
b. Which picture would you most prefer to look like?									
c. Which picture is most like other women your age?									
d. Which picture do you think most of the men around you would prefer women to look like?									

BODY SHAPE – QUESTIONS FOR MEN ONLY

829. We want to ask some questions about body shape. For the following questions, you can choose one of the pictures below. Under each picture is a number. Please use this number for your answer.

Please put an **X** under one of the numbers below:

	1	2	3	4	5	6	7	8	9
a. Which picture do you most look like right now?									
b. Which picture would you most prefer to look like?									
c. Which picture is most like other men your age?									
d. Which picture do you think most of the women around you would prefer men to look like?									

830.	How tall are you without shoes?<br>**IF DON'T KNOW, CODE 998 IN CENTIMETRES**	**Centimetres** ☐☐☐<br>**Feet** ☐ **Inches** ☐☐	
831.	How much do you weigh without clothes or shoes?<br>**IF DON'T KNOW, CODE 998 IN POUNDS**	**Pounds** ☐☐☐<br>**Kilos** ☐☐	

SECTION 9: SHOULD BE ADMINISTERED TO BOTH MALES AND FEMALES

SECTION 9: DIETARY PATTERNS, PSYCHOSOCIAL HEALTH AND GENERAL LIFESTYLES

SECTION 9A: DIETARY PATTERNS

901.	How often do you or someone else in your home prepare your meals at home?<br><br>1=NEVER 6=ONLY ON SUNDAYS<br>2=1 DAY/WEEK 7=2-3 DAYS/WEEK<br>3=EVERYDAY 8=ONLY ON SATURDAYS<br>4=WEEKENDS 9=OTHER SPECIFY____________________<br>5=3-4 DAYS/WEEK		☐	**IF CODE 1 SKIP TO Q908**
902.	If you cook your meals, what kinds of cooking methods do you use? ***(Check all that apply)***	**YES**	**NO**	
	a. FRYING	1	2	
	b. STEAMING	1	2	
	c. BAKING	1	2	
	d. GRILLING/BARBEQUE	1	2	
	e. BOILING	1	2	
	f. ROASTING	1	2	
	g. SMOKING	1	2	
903.	Which method do you use **most often**?<br><br>1=FRYING 2=Roasting 3=STEAMING OVER WATER, WITHOUT OIL<br>4=STEWING 5=BAKING 6=GRILLING/BARBEQUE 7=BOILING		☐	
904.	How often do you add salt to your meals at the table?<br><br>1=NEVER 2=RARELY 3=OCCASIONALLY 4=VERY OFTEN		☐	
905.	Do you do anything on a regular basis to control your salt intake?<br><br>1=YES 2=NO		☐	**IF CODE 2 SKIP TO Q907**
906.	What do you mainly do?<br><br>1=AVOID/MINIMIZE CONSUMPTION OF PROCESSED FOODS<br>2=LOOK AT THE SALT OR SODIUM LABELS ON FOOD<br>3=DO NOT ADD SALT AT THE TABLE<br>4=DO NOT ADD SALT WHEN COOKING<br>5=USE SPICES OTHER THAN SALT WHEN COOKING<br>6= AVOID EATING OUT<br>7= OTHER (SPECIFY) _______________		☐	
907.	What types of cooking oil do you usually use for frying/stewing at home?<br><br>01=PALM OIL 02= VEGETABLE OIL 03=SOYA BEAN OIL 04=OLIVE OIL<br>05=GROUNDNUT OIL 06= COCONUT OIL 07=PALM KERNEL OIL 8=LARD/ANIMAL FAT<br>9=CORNFLOUR OIL 10=SUNFLOWER OIL 11=DO NOT USE OIL AT ALL		☐☐	
908.	At what time of day do you usually eat your biggest/heavy meal?<br><br>1=MORNING 2=MIDDAY/AFTERNOON 3=EVENING		☐	
909.	Are there any specific dietary facts, or practices about Ghanaian foods that have not been mentioned in this survey?<br>1=YES<br>2=NO		☐	**IF CODE 2 SKIP TO Q911**
910.	Please explain any dietary facts, or practices about Ghanaian foods that have not been mentioned<br>____<br>____<br>____<br>____			

SECTION 9B: YOUR FOOD AND NUTRITION					
911. Which of the following food do you eat and how often do you eat them?					
**Food List**	**No. of times eaten in the past 7 days**	**Frequency of sources of food**			
		**Home**	**Chop bar**	**Street vendor**	**Restaurant**
**CEREAL-BASED PORRIDGES**					
Millet koko					
Maize koko					
Ricewater					
Oats					
Other (specify)____________________					
**CEREAL-BASED STAPLES**					
Rice-balls					
Banku/Akple/TZ/kenkey					
Plain rice					
Jollof rice					
Fried rice					
Waakye					
Other (specify)_________________________					
**TUBER & PLANTAIN BASED STAPLES**					
Fufu (all kinds)					
Ampesi (yam, cocoyam, plantain)					
Fried tubers (yam, plantain, cocoyam)					
Roasted tuber (yam, plantain, cocoyam					
Gari (*soakings, eba, or with beans*)					
Other (specify)____________________					
**SOUPS**					
Palm soup					
Light soup					
Groundnut soup					
Kontomire soup					
Okro soup (fresh)					
Okro soup (dry)					
Other (specify)____________________					
**STEWS/SAUCES**					
Tomatoes stew					
Palava sauce					
Garden egg stew					
Okro stew					
Beans stew *(with or without plantain)*					
Shito					
Ground pepper					
Other (specify)____________________					
**FATS & OILS**					
Red palm oil					
Vegetable (frytol, soy bean)					
Butter					
Margarine					
Lard & animal fat					
Other (specify)____________________					
**Continued….**					

Food List	No. of times eaten in the past 7 days	Frequency of sources of food			
		Home	Chop bar	Street vendor	Restaurant
**ANIMAL SOURCE FOODS**					
Livestock (goat, sheep, beef, pork)					
Poultry (chicken, duck, guinea fowl)					
Fish (tuna, herrings, salmon)					
Shell-fish (crab, lobster, shrimp, etc)					
Bush meat (grass cutter, antelope, etc)					
Egg					
Sausage					
Other (specify)____________________					
**BAKED/ROASTED/BOILED SNACKS**					
Bran/wheat bread					
Sugar/tea/butter bread					
Meat pie					
Cakes					
Cashew nut					
Roasted groundnuts					
Roasted maize					
Other (specify)____________________					
**FRIED SNACKS**					
Doughnut					
Chips (plantain , potato)					
Koose					
Chofi (Turkey tail)					
Other (specify)____________________					
**SOFT DRINKS**					
Minerals (Fanta, sprite coca cola)					
Malt drinks (Malta Guiness, vita malt)					
Fruit juices (Pure heaven, Don simon,)					
Energy drinks (eg. Lucozade, blue jeans etc.)					
Sweetened (Tampico, Kalyppo,)					
Other (specify)____________________					
**MILK AND DAIRY PRODUCTS**					
Milk					
Yoghurt /Fanmilk					
Cheese/Wagashie					
Other (specify)____________________					
**FRUITS**					
Orange					
Pineapple					
Banana					
Pawpaw					
Watermelon					
Mango					
Apple					
Grapes					
Avocado pear					
Other (specify)____________________					

YOUR FOOD AND NUTRITION CONTD.			
912.	Overall, when you think about the foods you ate over the past 12 months, would you say your diet was high, medium, or low in fat?<br>1=HIGH<br>2=MEDIUM<br>3=LOW		
913.	How often did you eat fast foods (like hamburgers, French fries, pizza, potato chips, fried rice, fried chicken, chips, etc)<br><br>1=NEVER 5=MORE THAN ONCE A WEEK<br>2= ONCE A YEAR 6= ONCE A WEEK<br>3=A FEW TIMES A YEAR 7=EVERYDAY<br>4= ONCE A MONTH		
914.	How many full meals do you usually eat each day?		
915.	How many snacks between meals do you usually eat each day?		
916.	Which one of the following statements best describes the food eaten in your household? Would you say you….<br><br>1=HAVE ENOUGH OF THE KINDS OF FOOD YOU EAT<br>2=HAVE ENOUGH BUT NOT ALWAYS WHAT YOU WANT TO EAT<br>3=SOMETIMES DO NOT HAVE ENOUGH TO EAT<br>4=OFTEN DO NOT HAVE ENOUGH TO EAT		`
917.	Do you regularly take vitamin and/or mineral supplements?<br>1=YES 2=NO		
918.	Do you regularly use any home remedies, herbal or natural products, or tonics for your health?<br>1=YES 2=NO		**IF CODE 2 SKIP TO Q920**
919.	What products do you take?<br>_______________________________________		
920.	How many times a week do you eat at fast food restaurants (like Papapye, Frankies, On the Run etc…)<br>1=_________ TIMES A WEEK<br>2=LESS THAN ONCE A WEEK<br>3=NEVER		
921.	How many times a week do you eat at other restaurants?<br>1=_________ TIMES A WEEK<br>2=LESS THAN ONCE A WEEK<br>3=NEVER		
922.	How often do you receive cooked food from friends/relatives/ neighbours as gifts?<br>1=EVERYDAY 4=ONLY ON SUNDAYS<br>2=3-4 DAYS A WEEK 5=NEVER<br>3=2-3 DAYS A WEEK 6=OTHER(SPECIFY) ___________________		

923.	At what types of stores do you usually get your food? ***(check <u>all</u> that apply)***. Now please tell me the store you shop at **<u>most often</u>**.	**YES**	**NO**	**Most Often Shop**	
	a. LARGE SUPERMARKET	1	2		
	b. NEIGHBOURHOOD GROCERY	1	2		
	c. CONVENIENCE STORE	1	2		
	d. FARM	1	2		
	e. THE BEACH (FOR FISH)	1	2		
	f. WAREHOUSE OR DISCOUNT STORE	1	2		
	g. SPECIALITY STORE (BUTCHER, BAKERY, ETC)	1	2		
	h. PRODUCE STAND/MARKET/WEEKEND MARKET	1	2		
	i. YOUR GARDEN	1	2		
	j. OTHER (SPECIFY)_______________________	1	2		

924.	How often do you usually shop for food?<br>1=EVERYDAY<br>2=A FEW TIMES A WEEK<br>3=ONCE A WEEK<br>4=LESS THAN ONCE A WEEK	☐	
925.	Is the store where you normally shop for food located in your neighbourhood?<br>1=YES 2=NO	☐	

SECTION 9C: PSYCHOSOCIAL HEALTH

These questions are about how you feel and how things have been with you during the past 4 weeks. For each question, please give the one answer that comes closest to the way you have been feeling. How much of the time during the past 4 weeks.

1=NONE OF THE TIME 2=A LITTLE OF THE TIME 3=SOME OF THE TIME 4=MOST OF THE TIME 5=ALL OF THE TIME

926.	a. DID YOU FEEL FULL OF LIFE AND BOUNCE?	a	
	b. HAVE YOU BEEN A VERY NERVOUS PERSON?	b	
	c. HAVE YOU FELT SO UNHAPPY AND NOT YOURSELF THAT NOTHING COULD CHEER YOU UP?	c	
	d. HAVE YOU FELT CALM AND PEACEFUL?	d	
	e. DID YOU HAVE A LOT OF ENERGY?	e	
	f. HAVE YOU FELT DOWNHEARTED AND LETDOWN?	f	
	g. DID YOU FEEL WORN OUT?	g	
	h. HAVE YOU FELT TIRED?	h	
	i. HAVE YOU BEEN A HAPPY PERSON?	i	
	j. HAVE YOU FELT WORTHLESS	j	
	k. HAVE YOU FELT HOPELESS?	k	
927.	In life people may/may not be content with life in general, I would like to ask about you? Do you feel content?<br>0=NOT AT ALL 1= A LITTLE BIT 2=MODERATELY 3= QUITE A BIT 4= EXTREMELY	☐	
928.	How do you see your health?<br>0=POOR 1=QUITE GOOD 2=GOOD 3=VERY GOOD 4=EXCELLENT	☐	

SECTION 9D: GENERAL LIFESTYLE

		YES	NO	
929.	Have you ever consumed any of these drinks that contain alcohol			**IF CODE 2, SKIP TO Q934**
	a. BEER/GUINNESS	1	2	
	b. WINE (NOT COMMUNION WINE)	1	2	
	c. AKPETESHIE	1	2	
	d. BRUKUTU	1	2	
	e. PITO	1	2	
	f. PALMWINE	1	2	
	g. GIN, WHISKEY OR OTHER SPIRITS	1	2	
	h. IRISH CREAM, AMARULA OR OTHER LIQUERS	1	2	
	i. LOCAL BITTERS (EG. AGYA APPIAH ETC…)	1	2	
	j. OTHER (SPECIFY)…………………………………….	1	2	

930.	Have you consumed alcohol in the last 30 days?<br>1=YES 2=NO	☐	**IF CODE 2 SKIP TO 932**
931.	Now think from now to this same time yesterday did you consume alcohol?<br>1=YES 2=NO	☐	
932.	How old were you when you first drunk alcohol?<br>DON'T KNOW………………………………………98	**AGE** ☐☐	
933.	How often in the past month have you had so much alcohol that you were really drunk?<br>1=NO, NEVER<br>2=NO, WAS NOT DRUNK IN PAST MONTH<br>3=YES, ONCE<br>4=YES, 2 TO 3 TIMES<br>5=YES, 4 TO 10 TIMES<br>6=YES, MORE THAN 10 TIMES<br>7=EVERYDAY<br>8=OTHER (SPECIFY)…………………………….	☐	

934.	Have you ever smoked tobacco or used smokeless tobacco?<br>1=YES 2=NO	☐	IF CODE 2 SKIP TO Q937
935.	Do you currently use (smoke, sniff or chew) any tobacco products such as cigarettes, cigars, pipes, chewing tobacco or snuff?<br>1=YES, DAILY 2=YES, BUT NOT DAILY 3=NO, NOT AT ALL	☐	IF CODE 2 OR 3 SKIP TO Q938
936.	For how long have you been smoking or using tobacco daily?<br>**[For interviewer: If less than one month- enter "00" for years and "00" for months]**	**YEARS** ☐☐ **MONTHS** ☐☐	
937.	Have you ever tried any kind of drug *(marijuana, etc)*?<br>1=YES 2=NO	☐	IF CODE 2 END SESSION

938. What have you tried? PROBE: what else?

	YES	NO
a. PILLS	1	2
b. MARIJUANA/WEE	1	2
c. COCAINE	1	2
d. PETROL SNIFFING	1	2
e. GLUE SNIFFING	1	2
f. PAINT SNIFFING	1	2
g. OTHER (SPECIFY)..	1	2
h. DON'T KNOW	1	2

939.	How old were you when you first tried one of these things?<br>98=DON'T KNOW	**AGE** ☐☐

END TIME FOR INTERVIEW **HOURS** ☐☐ **MINS** ☐☐

Do you have any question or comments for me?

....
....
....
....
....
....
....
....
....
....
....
....
....
....
....
....

WE HAVE COME TO THE END OF THE INTERVIEW, THANK YOU VERY MUCH FOR TAKING YOUR TIME TO ANSWER THESE QUESTIONS.
